# Supplementary material for: Canine Brachycephaly Is Associated with a Retrotransposon-Mediated Missplicing of SMOC2
Source: Curr Biol. 2017 Jun 5;27(11):1573–1584.e6. doi: 10.1016/j.cub.2017.04.057 (PMC5462623; doi:10.1016/j.cub.2017.04.057)
Supplement: Document S1. Figures S1–S7 and Tables S1, S2, and S4–S6 [file mmc1.pdf]

Current Biology, Volume *27*

Supplemental Information

Canine Brachycephaly Is Associated with a Retrotransposon-Mediated Missplicing of *SMOC2*

Thomas W. Marchant, Edward J. Johnson, Lynn McTeir, Craig I. Johnson, Adam Gow, Tiziana Liuti, Dana Kuehn, Karen Svenson, Mairead L. Bermingham, Michaela Drögemüller, Marc Nussbaumer, Megan G. Davey, David J. Argyle, Roger M. Powell, Sérgio Guilherme, Johann Lang, Gert Ter Haar, Tosso Leeb, Tobias Schwarz, Richard J. Mellanby, Dylan N. Clements, and Jeffrey J. Schoenebeck

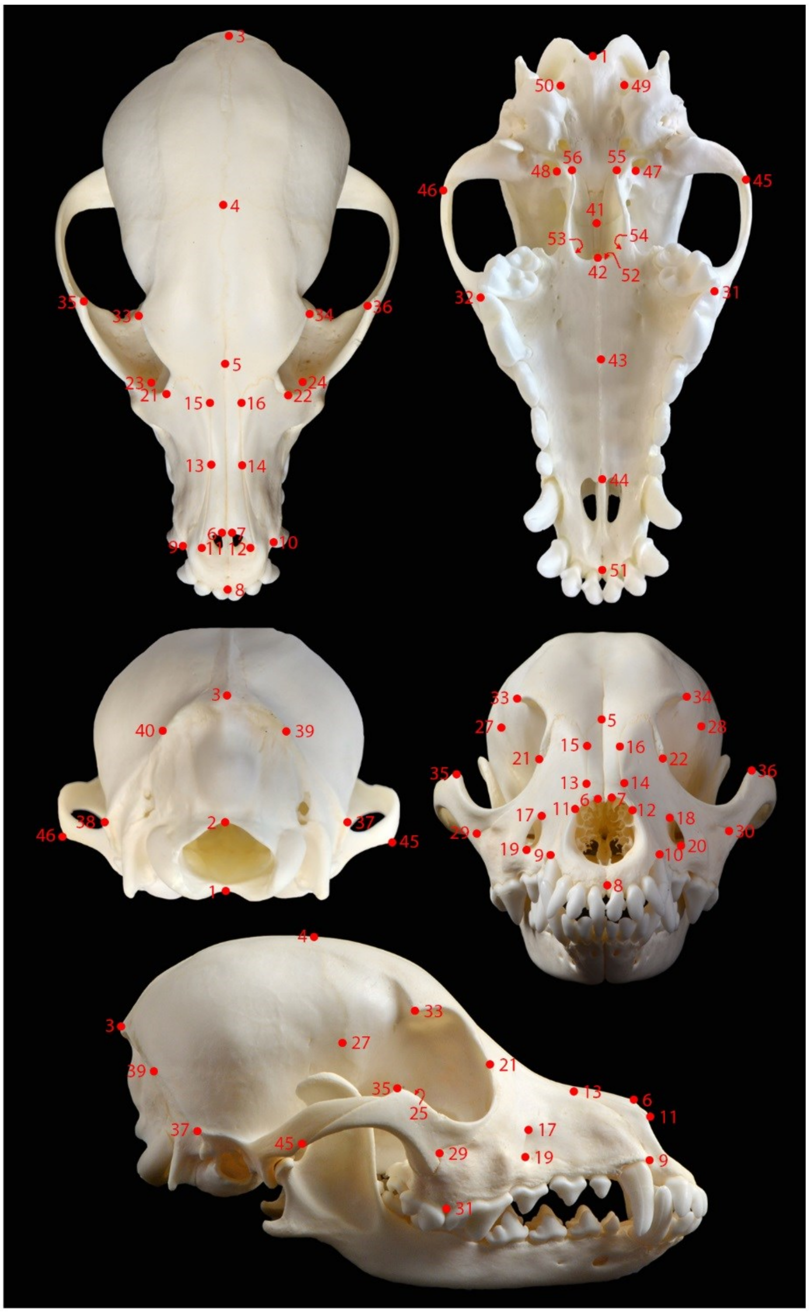

Skull Landmarks

1. Basion (Brainstem basis) *
2. Opisthion *
3. Inion *
4. Bregma *
5. Nasion * †
6/7. Internasal Suture (R+L) †
8. Prosthion †
9/10. Incisive-Maxilla Junction with Alveolus (R+L) †
11/12. Nasal Bone Process (R+L) †
13/14. Incisive-Maxilla-Nasal Junction (R+L) †
15/16. Frontal-Maxilla-Nasal Junction (R+L) †
17/18. Dorsal Aspect of the Infraorbital Foramen (R+L) †
19/20. Ventral Aspect of the Infraorbital Foramen (R+L) †
21/22. Frontal-Lacrimal-Maxilla Junction (R+L) †
23/24. Dorsal Aspect of the Maxillary Foramen (R+L)
25/26. Dorsal Aspect of the Ethmoid Foramina (R+L)
27/28. Pterion (R+L) *
29/30. Zygomatic Process of the Maxilla (R+L) †
31/32. Molar-Premolar Interalveolar Septa (R+L) †
33/34. Zygomatic Process of the Frontal Bone (R+L) *
35/36. Frontal Process of the Zygomatic Bone (R+L)
37/38. Nuchal Crest Flare (R+L) *
39/40. Asterion (R+L) *
41. Anterior Hirnstammbasis *
42. Posterior Nasal Spine
43. Maxilla-Palatine Junction at Midline †
44. Incisive-Maxilla Junction at Midline †
45/46. Ventral Temporal-Zygomatic Junction (R+L)
47/48. Rostral Aspect of the Foramen Ovale (R+L) *
49/50. Rostral Aspect of the Hypoglossal Canal (R+L) *
51. Intraoral Midline of Incisive Bone at Alveolus †
52. Midline of Vomer Dorsal to Nasal spine
53/54. Midline of Choana Dorsal to Posterior Nasal Spine (L+R)
55/56. Pterygoid Hamulus (L+R)

Mandible Landmarks

1/2. Dorsal Aspect of the Angular Process (L+R)
3/4. Lateral Aspect of the Condylar Process (L+R)
5/6. Medial Aspect of the Condylar Process (L+R)
7/8. Dorsal Aspect of the Mandibular Notch (L+R)
9/10. Dorso-Caudal Aspect of the Coronoid Process (L+R)
11/12. 3rd Molar Caudal-midline at Alveolus (L+R)
13/14. Ventral Aspect of the Masseteric Fossa (L+R)
15/16. 1st Molar Lateral-midline at Alveolus (L+R)
17/18. Canine Tooth Caudal-midline at Alveolus (L+R)
19/20. Caudal Aspect of Caudal Mental Foramina (L+R)
21/22. Caudal Aspect of Middle Mental Foramina (L+R)
23/24. Caudal Aspect of Rostral Mental Foramina (L+R)
25/26. Ventral Aspect of Inter-Mandibular Joint (L+R)
27/28. Rostral Aspect of the Mandibular Foramen (L+R)
29/30. Mid-Coronoid Process (L+R)

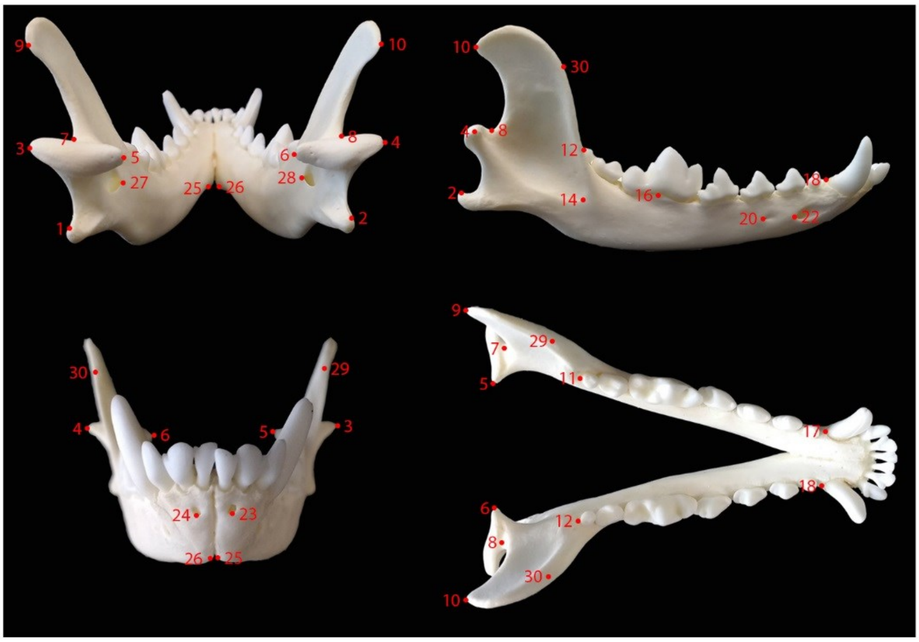

Figure S1. Landmarks of the skull and Mandible. Related to Figure 1, 2 and S3. Images illustrate the position of landmarks placed on skull isosurfaces. Skull images clockwise from top-left: Dorsal, ventral, rostral, lateral (right side) and caudal views. Mandible images clockwise from top-left: Caudal, lateral (right side), dorsal and rostral views. The canine anatomic denominations correspond to a human skull as follows: Rostral = Anterior, Caudal = Posterior, Ventral = Inferior, Dorsal = Superior. Skull landmarks are subdivided into neurocranium (*) and viscerocranium (†) datasets.

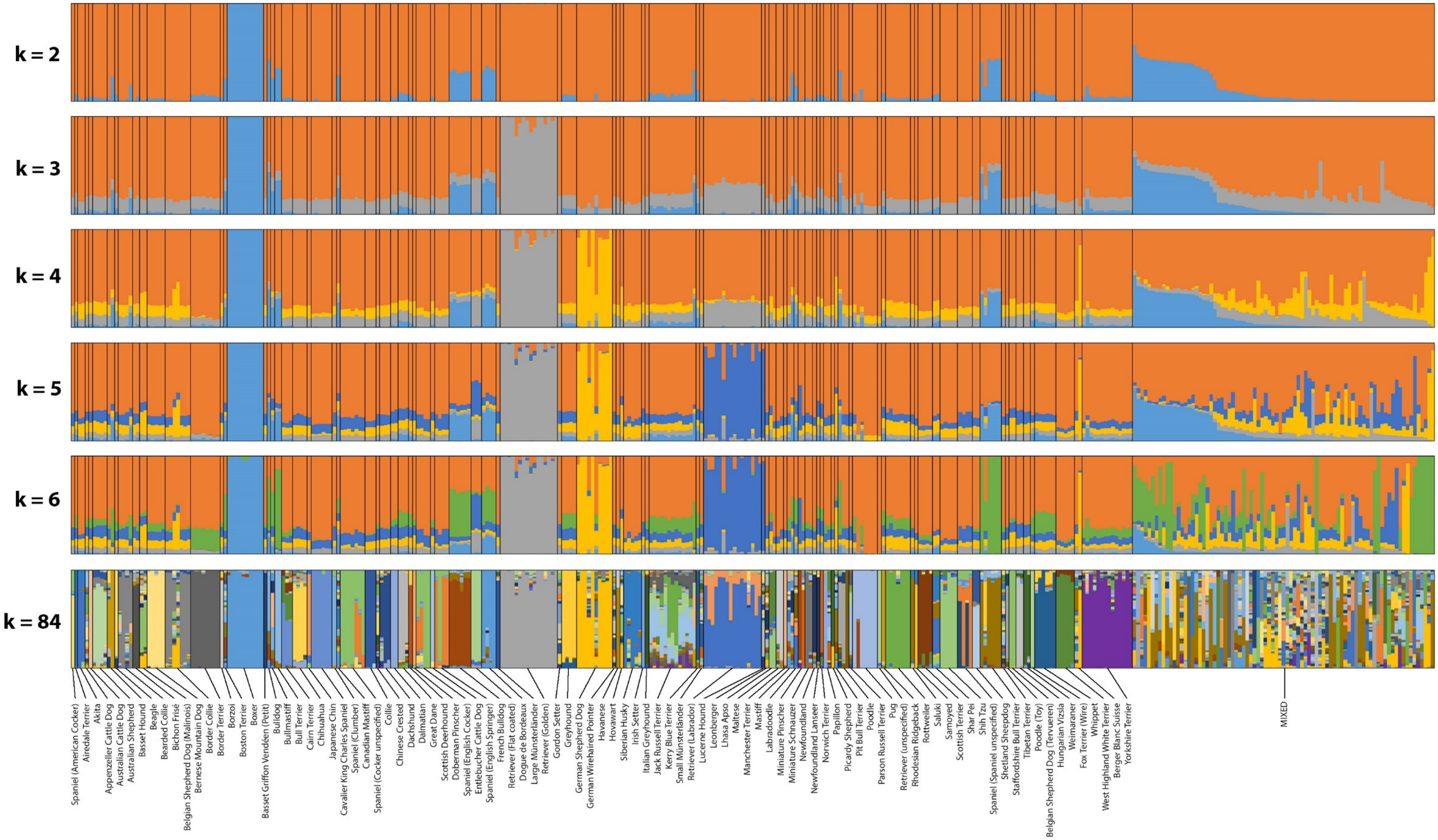

Figure S2. The genomic structure confirms the correct assignment of breed identities and infers the breed composition of mixed-breed dogs. Related to Figure 1 and 3. Population structure of all 374 individuals in this study. Each vertical bar represents an individual dog. Dogs are ordered by breed (vertical black lines). Vertical bars are split into different colours depending on the proportional membership of an individual dog to each cluster.

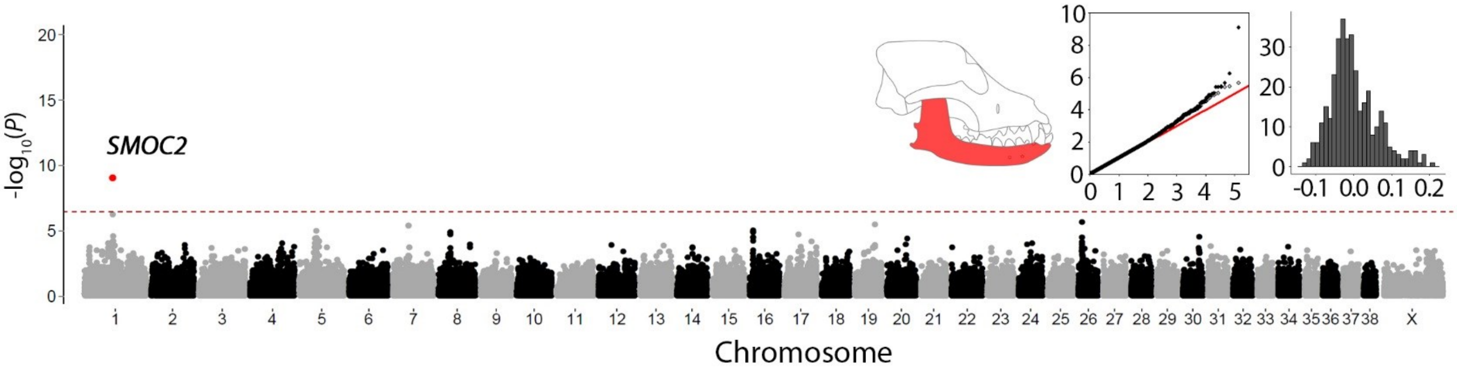

Figure S3. Variation in mandible shape is associated with a QTL on CFA1. Related to Figure 2 and S1. Manhattan plot for mandible PC1 was generated using a linear mixed model in GEMMA software. The mandible dataset was regressed using the neurocranium centroid to remove the effects of allometry. Sex and five principal components computed from SNP data were used as covariates. SNPs remaining significant following a Bonferroni correction (3.6×10^{-7}, dashed line) are coloured red. Significant SNPs are summarised in Table S3. Inserts: skull schematics indicate the origins of landmarks used for dataset. Expected (x-axis) and observed (y-axis) $-\log_{10}(P)$ values are plotted for all SNPs (black circles) and pruned SNPs (grey circles). Histogram depicts the frequency (y-axis) of mandible PC1.

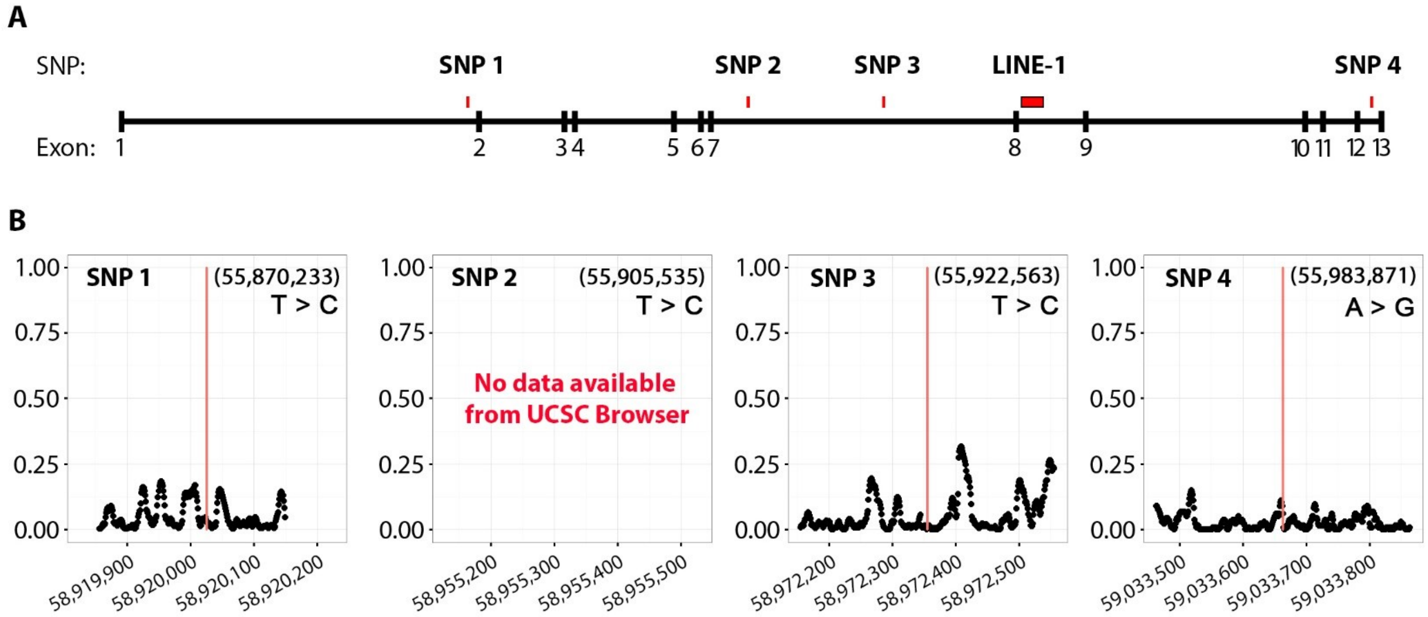

Figure S4. Remaining intronic candidate SNPs are poorly conserved across species. Related to Table 2. (A) All four remaining SNPs in the viscerocranium PC1 critical interval following filtering are located within the *SMOC2* gene. (B) Coordinates of each SNP (listed as CanFam3.1 in brackets) are converted to CanFam2.0 and used to plot PhastCon scores (y-axis) for 200 bp in both directions from the SNP (x-axis). PhastCon scores were downloaded from the UCSC Table Browser. No PhastCon scores were available from the UCSC Table Browser for SNP2. From the available data, no SNPs reside in highly conserved positions across dog, human, mice and rat genomes. Alleles are written as Ancestral > Derived.

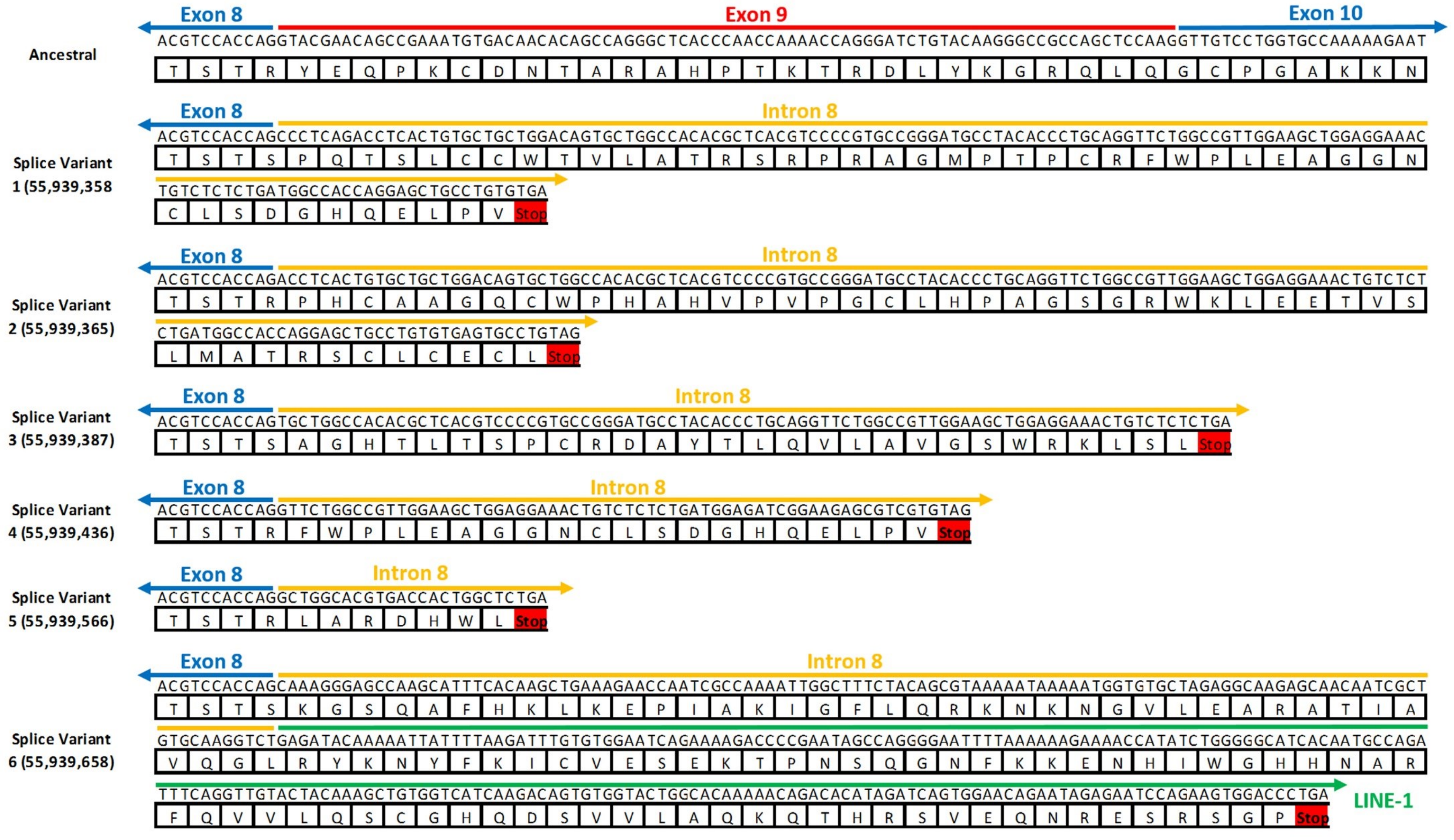

Figure S5. *SMOC2* Isoform Sequences. Related to Figure 4. mRNA and amino acid sequences for the canonical (ancestral) *SMOC2* transcript and the six isoforms identified in dogs that are carriers of the LINE-1 element. Base pair positions in brackets indicate the position of the first nucleotide of intron 8 to be included in the mature mRNA transcript. All isoforms are predicted to code for premature stop codons.

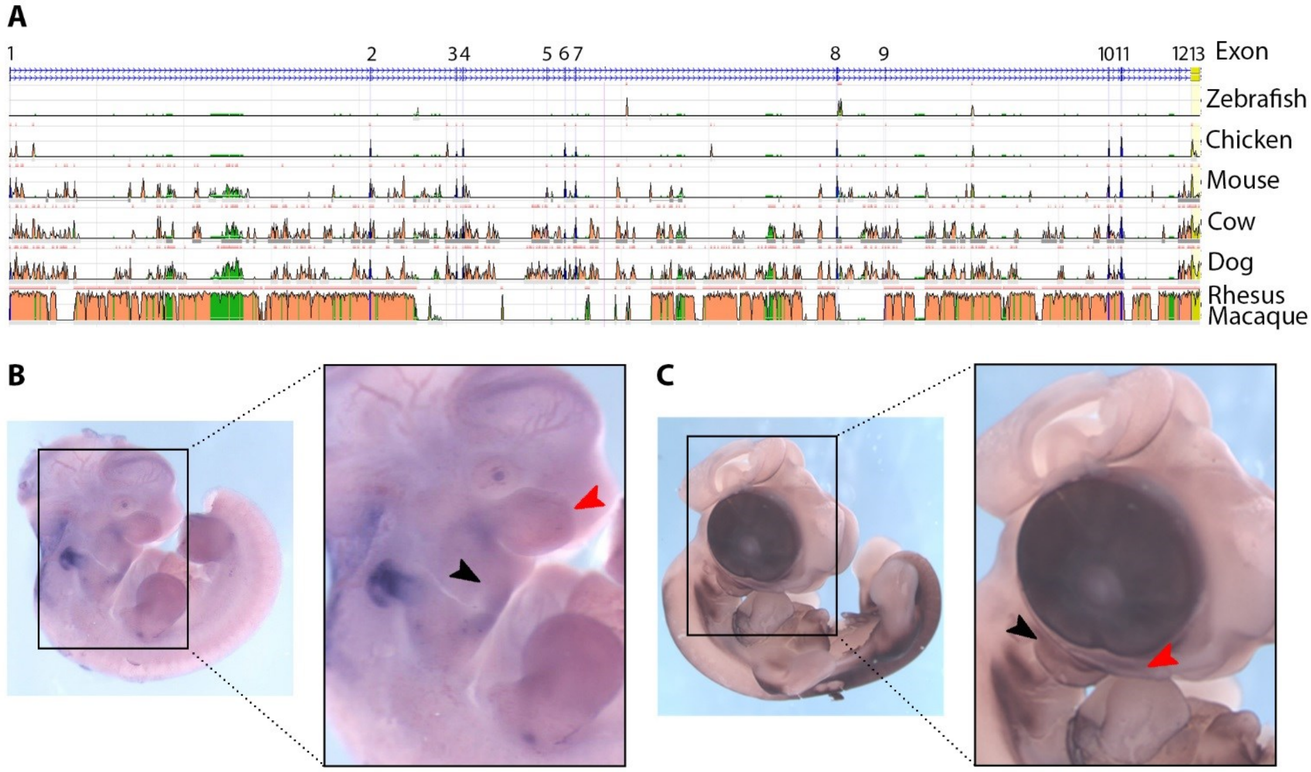

Figure S6. Evolutionary Conservation of *SMOC2*. Related to Figure 3. The Evolutiponary Conserved Regions of human *SMOC2* spanning chr6:168,851,429-169,078,272 (hg19 construct) are poorly conserved in the zebrafish, chicken and mouse compared to the dog, cow and rhesus monkey (A). Despite this, *Smoc2* mRNA is expressed in the first pharyngeal arch of the mouse and chicken. Whole mount in situ hybridisations for *Smoc2* in mouse (**B**) and chicken (**C**) embryos. *Smoc2* expression is observed in the mandibular process (black arrow) and maxillary process (red arrow) of the first pharyngeal arch.

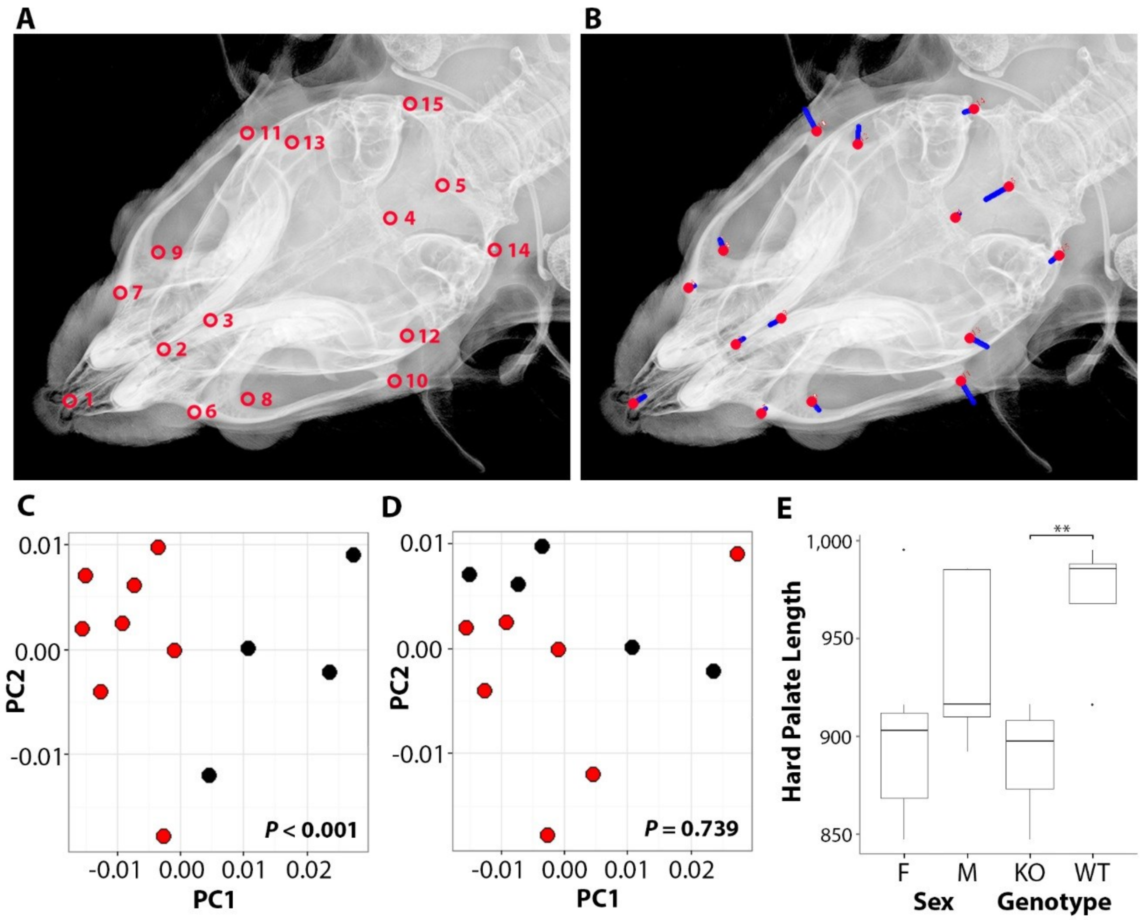

Figure S7. *Smoc2* null mice have dysmorphic skulls. Related to Figure 4. (A) Dorsoventral radiographs of mice aged 13 weeks indicating the position of fifteen landmarks across the skull. (B) Lollipop diagram denotes the mean position of landmarks (red circles) and the extent and direction of landmark variation for whole head PC1 (blue bars). The whole skull PC1 values for *Smoc2* null (red) and control mice (black) segregate significantly by (C) genotype but not (D) sex.

Abbreviation	Breed Name	Kennel Club (UK) Recognised	Viscerocranium Dataset	Mandible Dataset
ACKR	Spaniel (American Cocker)	Yes	1	1
AIRT	Airedale Terrier	Yes	1	1
AKIT	Akita	Yes	2	2
AUCD	Australian Cattle Dog	Yes	1	1
AUSS	Australian Shepherd	Yes	4	4
BASS	Basset Hound	Yes	2	2
BEAG	Beagle	Yes	1	1
BERD	Bearded Collie	Yes	4	4
BICH	Bichon Frisé	Yes	2	1
BMAL	Belgian Shepherd Dog (Malinois)	Yes	2	2
BMD-	Bernese Mountain Dog	Yes	5	5
BORD	Border Collie	Yes	7	6
BORT	Border Terrier	Yes	8	7
BORZ	Borzoi	Yes	1	1
BOST	Boston Terrier	Yes	1	1
BOX-	Boxer	Yes	10	10
BRGR	Basset Griffon Vendéen (Petit)	Yes	1	1
BULD	Bulldog	Yes	1	1
BULM	Bullmastiff	Yes	1	1
BULT	Bull Terrier	Yes	2	2
CAIR	Cairn Terrier	Yes	3	2
CHIH	Chihuahua	Yes	4	3
CHIN	Japanese Chin	Yes	1	1
CKCS	Cavalier King Charles Spaniel	Yes	6	6
CLSP	Spaniel (Clumber)	Yes	1	1
COLL	Collie	Yes	3	3
CRES	Chinese Crested	Yes	1	0
DACH	Dachshund	Yes	3	2
DALM	Dalmatian	Yes	2	2
DANE	Great Dane	Yes	3	3
DEER	Scottish Deerhound	Yes	1	1
DOBP	Doberman Pinscher	Yes	1	1
ECKR	Spaniel (English Cocker)	Yes	3	3
ENTL	Entlebucher Cattle Dog	Yes	1	1
ESSP	Spaniel (English Springer)	Yes	4	3
FBUL	French Bulldog	Yes	6	6
FCR-	Retriever (Flat coated)	Yes	3	3
FMAS	Dogue de Bordeaux	Yes	4	4
GMLD	Large Münsterländer	Yes	1	1
GOLD	Retriever (Golden)	Yes	16	15
GORD	Gordon Setter	Yes	1	1
GREY	Greyhound	Yes	4	3
GSD-	German Shepherd Dog	Yes	10	10
GWHP	German Wirehaired Pointer	Yes	1	1
HAVA	Havanese	Yes	1	1
HOVA	Hovawart	Yes	1	1
HUSK	Siberian Husky	Yes	5	5
IRSE	Irish Setter	Yes	1	1
ITGY	Italian Greyhound	Yes	1	1
JACK	Jack Russell Terrier	Yes	13	13
KERY	Kerry Blue Terrier	Yes	1	1
KMLD	Small Münsterländer	Yes	1	1
LAB-	Retriever (Labrador)	Yes	16	14
LEON	Leonberger	Yes	1	1
LHSA	Lhasa Apso	Yes	2	2
MALT	Maltese	Yes	2	2
MANT	Manchester Terrier	Yes	1	1
MAST	Mastiff	Yes	2	2

MPIN	Miniature Pinscher	Yes	2	2
MSNZ	Miniature Schnauzer	Yes	2	2
NEWF	Newfoundland	Yes	1	1
NOWT	Norwich Terrier	Yes	1	1
PAPI	Papillon	Yes	2	1
PICA	Picardy Shepherd	Yes	1	1
PRUS	Parson Russell Terrier	Yes	1	1
PUG-	Pug	Yes	7	7
RHOD	Rhodesian Ridgeback	Yes	1	1
ROTT	Rottweiler	Yes	7	7
SALU	Saluki	Yes	1	1
SAMO	Samoyed	Yes	1	1
SCOT	Scottish Terrier	Yes	4	3
SHAR	Shar Pei	Yes	2	2
SHIH	Shih Tzu	Yes	5	5
SSHP	Shetland Sheepdog	Yes	2	2
STAF	Staffordshire Bull Terrier	Yes	6	6
TIBT	Tibetan Terrier	Yes	1	1
TPOO	Poodle (Toy)	Yes	1	1
TURV	Belgian Shepherd Dog (Tervueren)	Yes	2	2
VIZS	Hungarian Vizsla	Yes	2	2
WEIM	Weimaraner	Yes	2	2
WFOX	Fox Terrier (Wire)	Yes	1	1
WHIP	Whippet	Yes	6	6
WHWT	West Highland White Terrier	Yes	5	3
YORK	Yorkshire Terrier	Yes	14	12
APPH	Appenzeller Cattle Dog	No	1	0
CKSP	Spaniel (Cocker unspecified)	No	7	7
CMAS	Canadian Mastiff	No	1	1
LBDO	Labradoodle	No	1	1
LCHO	Lucerne Hound	No	1	1
MIXED	Mixed Breed	No	83	83
NEWL	Newfoundland Lanseer	No	1	1
PITB	Pit Bull Terrier	No	1	1
POOD	Poodle	No	3	3
RTVR	Retriever (unspecified)	No	1	1
SPSP	Spaniel (Spaniel unspecified)	No	4	4
WSSD	Berger Blanc Suisse	No	2	2
		**Total:**	374	355

Table S1. Breed Demographics and Abbreviations. Related to Figure 1. Summary of the breed designation of all 374 individuals in the viscerocranium dataset and 355 individuals in the mandible dataset. Eighty-four breeds are recognised by the Kennel Club (UK).

Dataset	CFA	SNP	Position	Candidate Gene	Allele	Beta	*P*-value	Reference
Viscerocranium	1	BICF2P250912	55,983,871	***SMOC2***	G > A	-7.04E-02	1.91E-20	
		BICF2P1251361	55,862,036	***SMOC2***	C > T	-3.37E-02	1.39E-09	
		BICF2P1290166	56,132,332	*SMOC2*	A > G	-3.31E-02	1.64E-07	
Mandible	1	BICF2P250912	55,983,871	***SMOC2***	G > A	2.37E-02	8.43E-10	
Neurocranium	3	TIGRP2P56799_rs8666557	91,103,945	*LCORL/NCAPG*	G > T	1.02E+01	3.64E-09	[S1]
		BICF2G630361702	91,114,590	*LCORL/NCAPG*	G > A	1.03E+01	8.31E-09	
	7	BICF2S23352941	43,719,549	***SMAD2***	A > G	-1.11E+01	5.71E-13	[S1, S2, S3]
		BICF2S23762784	43,702,273	***SMAD2***	G > A	-1.03E+01	8.17E-12	
		BICF2P884876	43,569,230	*SMAD2*	G > C	-7.22E+00	1.65E-07	
		BICF2S23719803	43,865,905	*SMAD2*	T > C	7.64E+00	2.60E-07	
	10	G580f46S240	8,183,593	***HMGA2***	C > T	-1.41E+01	3.06E-15	[S1, S2, S3]
		BICF2P776298	8,454,499	*HMGA2*	A > G	-1.01E+01	4.36E-13	
		BICF2S23211087	7,993,147	*HMGA2*	T > C	-1.00E+01	2.77E-11	
		BICF2P283272	7,996,770	*HMGA2*	G > A	-1.00E+01	2.77E-11	
		BICF2P462764	8,772,534	*HMGA2*	T > C	-9.73E+00	3.78E-11	
		BICF2P1118840	7,957,984	*HMGA2*	T > C	-9.97E+00	3.86E-11	
		BICF2P263265	8,162,459	***HMGA2***	T > C	-9.24E+00	1.32E-09	
		BICF2P718692	8,070,103	*HMGA2*	C > T	9.40E+00	4.76E-09	
		BICF2S24410053	7,892,540	*HMGA2*	C > A	-8.21E+00	8.81E-08	
		BICF2P712340	7,884,978	*HMGA2*	T > C	8.60E+00	9.34E-08	
		BICF2S23633421	8,085,469	*HMGA2*	T > C	8.43E+00	1.01E-07	
		BICF2P797505	8,418,771	*HMGA2*	G > C	8.02E+00	1.50E-07	
		BICF2S23128717	8,779,119	*HMGA2*	G > A	7.04E+00	1.89E-07	
	15	BICF2P355320	41,257,020	***IGF1***	C > T	-1.63E+01	1.73E-19	[S1, S2, S3]
		BICF2P971192	41,221,438	***IGF1***	A > G	1.18E+01	4.38E-18	
		BICF2P893982	41,216,597	***IGF1***	A > G	1.16E+01	1.63E-17	
		BICF2P885652	41,232,547	***IGF1***	C > T	-1.04E+01	1.18E-15	
		BICF2P1107998	41,247,955	***IGF1***	C > T	-1.02E+01	1.30E-15	
		BICF2P355319	41,256,949	***IGF1***	A > G	1.01E+01	2.66E-15	
		BICF2P67088	41,206,514	***IGF1***	C > A	9.95E+00	2.23E-12	
		BICF2P117496	41,250,986	***IGF1***	C > T	9.45E+00	6.12E-11	
		BICF2P1100250	41,263,373	*IGF1*	A > C	9.12E+00	6.12E-11	
		BICF2P1292476	41,177,094	***IGF1***	T > C	7.27E+00	3.27E-08	
		BICF2P577974	41,314,290	*IGF1*	G > A	-6.88E+00	3.15E-07	
	18	BICF2S23615757	20,272,961	*FGF4* retrogene	A > C	-1.14E+01	3.31E-08	[S1, S4]
		BICF2S2323033	20,310,833	*FGF4* retrogene	T > G	-8.90E+00	2.72E-07	

Table S2. Significant SNPs. Related to Figure 2, S3 and Table 1. All significant SNPs detected in viscerocranium PC1, mandible PC1 and neurocranium centroid size datasets (Figure 2, Supplementary Figure 4). Candidate genes for each locus are listed. Gene names are highlighted in bold for intragenic SNPs.

Table S3. CFA1 Critical Interval Haplotype Summary.

See Supp_Table_3_Haplotype_Summary_2017.xlsx which is available for download.

Individual	Criteria	SnpSift (SNPs/INDELS)	Pindel (Structural variants)
American Bulldog	Homozygous Reference (Derived)	✓	✓
Brussels Griffon	Homozygous Reference (Derived)	✓	✓
French Bulldog	Homozygous Reference (Derived)	✓	✓
Mixed	Homozygous Reference (Derived)	✓	✓
Pug	Homozygous Reference (Derived)	✓	✓
Pug	Homozygous Reference (Derived)	✓	✓
Shih Tzu	Homozygous Reference (Derived)	✓	✓
Airedale Terrier	Homozygous Variant (Ancestral)	✓	✓
Italian Greyhound	Homozygous Variant (Ancestral)	✓	✓
Saluki	Homozygous Variant (Ancestral)	✓	✓
Whippet	Homozygous Variant (Ancestral)	✓	✓
Wolf	Homozygous Variant (Ancestral)	✓	X
Wolf	Homozygous Variant (Ancestral)	✓	X
Wolf	Homozygous Variant (Ancestral)	✓	X
Wolf	Homozygous Variant (Ancestral)	✓	X

Table S4. **Filtering Criteria.** Related to Table 2. Criteria for hard filtering variants within the critical interval (CFA1:55,850,299 – 56,037,676) were determined by the presence or absence of the derived twelve-SNP haplotype. A variant must satisfy each criteria in order to remain as a candidate for SnpSift or Pindel filtering.

Transcript	Silent Variant (55,939,143)	Splice Donor			Splice Acceptor			RNAseq Transcript Count								
		Position	Feature	Sequence	Sequence	Feature	Position	BULD	FBUL	PUG-	CKCS	CKCS	PAPI	ITGY	WHIP	YORK
Canonical (Ancestral)	AAT GA**C/T** AAC	55,939,268	Exon 8	TCC ACC AG **gt**aagg	tcgt**ag** G TAC GAA	Exon 9	55,947,842	224	84	116	258	1012	361	493	644	911
Splice Variant 1	AAT GA**T** AAC	55,939,268	Exon 8	TCC ACC AG **gt**aagg	gctc**ag** C CCT CAG	Intron 8	55,939,358	5 (2.23)	0 (0)	0 (0)	0 (0)	0 (0)	0 (0)	0 (0)	0 (0)	0 (0)
Splice Variant 2	AAT GA**T** AAC	55,939,268	Exon 8	TCC ACC AG **gt**aagg	cctc**ag** A CCT CAC	Intron 8	55,939,365	0 (0)	0 (0)	0 (0)	0 (0)	0 (0)	2 (0.55)	0 (0)	0 (0)	0 (0)
Splice Variant 3	AAT GA**T** AAC	55,939,268	Exon 8	TCC ACC AG **gt**aagg	ggac**ag** T GCT GGC	Intron 8	55,939,387	0 (0)	0 (0)	0 (0)	0 (0)	2 (0.19)	0 (0)	0 (0)	0 (0)	0 (0)
Splice Variant 4	AAT GA**T** AAC	55,939,268	Exon 8	TCC ACC AG **gt**aagg	ctgc**ag** G TTC TGG	Intron 8	55,939,436	87 (38.83)	17 (20.23)	43 (37.06)	9 (3.48)	40 (3.95)	13 (3.60)	0 (0)	0 (0)	1 (0.10)
Splice Variant 5	AAT GA**T** AAC	55,939,268	Exon 8	TCC ACC AG **gt**aagg	atac**ag** G CTG GCA	Intron 8	55,939,566	2 (0.89)	1 (1.19)	4 (3.44)	1 (0.38)	3 (0.29)	0 (0)	2 (0.40)	0 (0)	0 (0)
Splice Variant 6	AAT GA**T** AAC	55,939,268	Exon 8	TCC ACC AG **gt**aagg	tttc**ag** C AAA GGG	Intron 8	55,939,658	20 (8.92)	7 (8.33)	11 (9.48)	1 (0.38)	5 (0.49)	0 (0)	0 (0)	0 (0)	0 (0)

Table S5. Splice Site Sequences for *SMOC2* Isoforms. Related to Figure 4. Upper-case letters indicate sequence included in mRNA. Lower-case letters indicate intron sequence that is not included in mature mRNA transcripts. Bold letters are the consensus splice donor (gt) and splice acceptor (ag) sites. Three dogs for each *SMOC2* LINE-1 genotype status were used for RNAseq analysis – homozygous derived (Bulldog, French Bulldog, Pug) heterozygous (Cavalier King Charles Spaniel x2, Papillion) and homozygous ancestral (Italian Greyhound, Whippet, Yorkshire Terrier). The percentage of splice variant read count of the canonical transcript count for each dog are given in brackets.

Individual	Breed	*SMOC2* Genotype	Silent Variant (55,939,143)	
			T-allele	C-allele
1	BULD	DD	100%	
2	FBUL	DD	100%	
3	PUG-	DD	100%	
4	PAPI	AD	100%	
5	CKCS	AD	24%	76%
6	CKCS	AD	25%	75%
7	ITGY	AA	100%	
8	WHIP	AA	100%	
9	YORK	AA	54%	46%

Table S6. Allele-specific Expression of *SMOC2*. Related to Figure 4. RNAseq data was generated for nine dogs for the homozygous derived (D/D), heterozygous (AD) and homozygous ancestral (AA) genotypes of *SMOC2*. The sequencing of the C/T silent mutation in exon 8 is given as a percentage of the total read counts for the 55,939,143 base position. The LINE-1 element is found on a T-allele which suggests the decreased abundance of transcripts from the LINE-1 carrying haplotype.

Supplemental References

S1. Hayward, J. J., Castelhano, M. G., Oliveira, K. C., Corey, E., Balkman, C., Baxter, T. L., Casal, M. L., Sharon A Center, Fang, M., Garrison, S. J., et al. (2016). Complex disease and phenotype mapping in the domestic dog. Nat Commun *7*, 10460.

S2. Boyko, A. R., Quignon, P., Li, L., Schoenebeck, J. J., Degenhardt, J. D., Lohmueller, K. E., Zhao, K., Brisbin, A., Parker, H. G., vonHoldt, B. M., et al. (2010). A simple genetic architecture underlies morphological variation in dogs. PLoS Biol *8*, e1000451.

S3. Rimbault, M., Beale, H. C., Schoenebeck, J. J., Hoopes, B. C., Allen, J. J., Kilroy-Glynn, P., Wayne, R. K., Sutter, N. B., and Ostrander, E. A. (2013). Derived variants at six genes explain nearly half of size reduction in dog breeds. Genome Res *23*, 1985–1995.

S4. Parker, H. G., vonHoldt, B. M., Quignon, P., Margulies, E. H., Shao, S., Mosher, D. S., Spady, T. C., Elkahloun, A., Cargill, M., Jones, P. G., et al. (2009). An expressed fgf4 retrogene is associated with breed-defining chondrodysplasia in domestic dogs. Science *325*, 995–998.
