# Supplementary material for: Canine Brachycephaly Is Associated with a Retrotransposon-Mediated Missplicing of SMOC2
Source: Curr Biol. 2017 Jun 5;27(11):1573–1584.e6. doi: 10.1016/j.cub.2017.04.057 (PMC5462623; doi:10.1016/j.cub.2017.04.057)
Supplement: Document S2. Article plus Supplemental Information [file mmc3.pdf]

Current Biology

Article

Canine Brachycephaly Is Associated with a Retrotransposon-Mediated Missplicing of *SMOC2*

Highlights

- A population-based genetics study of dogs that required diagnostic imaging
- Resolution of a QTL associated with face length reduction (brachycephaly)
- Association of brachycephaly with a retrotransposon that disrupts *SMOC2* splicing
- The *SMOC2* locus explains up to 36% of face length variation in dogs

Authors

Thomas W. Marchant, Edward J. Johnson, Lynn McTeir, ..., Richard J. Mellanby, Dylan N. Clements, Jeffrey J. Schoenebeck

Correspondence

jeff.schoenebeck@roslin.ed.ac.uk

In Brief

Uncovering the molecular basis of dogs' vastly diverse skull shapes requires individualized approaches to morphometrics and genotyping. Using data from veterinary referral patients, Marchant et al. shed light on a large effect locus that is responsible for face length reduction (brachycephaly).

Marchant et al., 2017, Current Biology *27*, 1573–1584
June 5, 2017 © 2017 The Authors. Published by Elsevier Ltd.
http://dx.doi.org/10.1016/j.cub.2017.04.057

Current Biology
Article

Canine Brachycephaly Is Associated with a Retrotransposon-Mediated Missplicing of *SMOC2*

Thomas W. Marchant,[1] Edward J. Johnson,[1] Lynn McTeir,[1] Craig I. Johnson,[1] Adam Gow,[1] Tiziana Liuti,[1] Dana Kuehn,[2] Karen Svenson,[3] Mairead L. Bermingham,[4] Michaela Drögemüller,[5] Marc Nussbaumer,[6] Megan G. Davey,[1] David J. Argyle,[1] Roger M. Powell,[7] Sérgio Guilherme,[8] Johann Lang,[9] Gert Ter Haar,[10] Tosso Leeb,[5] Tobias Schwarz,[1] Richard J. Mellanby,[1] Dylan N. Clements,[1] and Jeffrey J. Schoenebeck[1,11,*]
[1]Royal (Dick) School of Veterinary Studies and Roslin Institute, University of Edinburgh, Easter Bush, Midlothian EH25 9RG, UK
[2]Friendship Hospital for Animals, Washington, DC 20016, USA
[3]The Jackson Laboratory, Bar Harbor, ME 04609, USA
[4]Institute of Genetics and Molecular Medicine, University of Edinburgh, Edinburgh EH4 2XU, UK
[5]Institute of Genetics, University of Bern, 3001 Bern, Switzerland
[6]Naturhistorisches Museum, Bernastrasse 15, 3005 Bern, Switzerland
[7]Powell Torrance Diagnostic Services, Manor Farm Business Park, Higham Gobion, Hertfordshire SG5 3HR, UK
[8]Davies Veterinary Specialists, Manor Farm Business Park, Higham Gobion, Hertfordshire SG5 3HR, UK
[9]Division of Clinical Radiology, Department of Clinical Veterinary Medicine, University of Bern, 3001 Bern, Switzerland
[10]Department of Clinical Sciences and Services, Royal Veterinary College, Hertfordshire AL9 7TA, UK
[11]Lead Contact
*Correspondence: jeff.schoenebeck@roslin.ed.ac.uk
http://dx.doi.org/10.1016/j.cub.2017.04.057

SUMMARY

In morphological terms, "form" is used to describe an object's shape and size. In dogs, facial form is stunningly diverse. Facial retrusion, the proximodistal shortening of the snout and widening of the hard palate is common to brachycephalic dogs and is a welfare concern, as the incidence of respiratory distress and ocular trauma observed in this class of dogs is highly correlated with their skull form. Progress to identify the molecular underpinnings of facial retrusion is limited to association of a missense mutation in *BMP3* among small brachycephalic dogs. Here, we used morphometrics of skull isosurfaces derived from 374 pedigree and mixed-breed dogs to dissect the genetics of skull form. Through deconvolution of facial forms, we identified quantitative trait loci that are responsible for canine facial shapes and sizes. Our novel insights include recognition that the *FGF4* retrogene insertion, previously associated with appendicular chondrodysplasia, also reduces neurocranium size. Focusing on facial shape, we resolved a quantitative trait locus on canine chromosome 1 to a 188-kb critical interval that encompasses *SMOC2*. An intronic, transposable element within *SMOC2* promotes the utilization of cryptic splice sites, causing its incorporation into transcripts, and drastically reduces *SMOC2* gene expression in brachycephalic dogs. *SMOC2* disruption affects the facial skeleton in a dose-dependent manner. The size effects of the associated *SMOC2* haplotype are profound, accounting for 36% of facial length variation in the dogs we tested. Our data bring new focus to *SMOC2* by highlighting its clinical implications in both human and veterinary medicine.

INTRODUCTION

The mammalian skull is an architectural wonder that illustrates the intertwined relationship of form and function. The skull facilitates ingestion and respiration, provides protection for the brain, and houses the visual, auditory, and olfactory systems. The skull also functions in communication, defense, and reproductive behaviors. The pressures of natural selection have ensured that the skull, a composite of bones, is multifunctional and is physically matched to the environmental challenges it experiences.

Human intervention through domestication and artificial selection has largely displaced the influence of natural selection on form and function across domestic species. The most profound effects of human intervention across all terrestrial species can be observed among skulls of the domestic dog, *Canis familiaris* [1]. Centuries of selective breeding has resulted in a broad radiation in skull form [2] whereas restraints on function have been relaxed.

Some subpopulations of dogs display morphologies that are highly reminiscent of human craniofacial anomalies, such as brachycephaly-type craniosynostosis and midface hypoplasia. In both species, brachycephaly and midface hypoplasia are risk factors for developing severe morbidities, including respiratory [3], gastrointestinal [3, 4], ear- and eye-related morbidities [3, 5], and neurological abnormalities [6]. Due to their rarity and complex clinical presentation, most human patients with brachycephaly will never receive a genetic diagnosis [7]. Conversely, dogs represent abundant examples of morphologically varied skull shapes.

Previous investigations of canine head shape using genome-wide association studies (GWASs) and selective sweep mapping highlighted an association between canine chromosome (CFA) 1 and brachycephaly [8–10]. In a binary design of brachycephalic

This is an open access article under the CC BY license (http://creativecommons.org/licenses/by/4.0/).

versus non-brachycephalic pedigree dogs, Bannasch et al. [11] established a 296-kb haplotype that encompassed the thrombospondin 2 (*THBS2*) gene. This study did not identify causal genetic variants, and the effects of this locus on gene expression were not assessed [11]. Elsewhere, measurements and geometric morphometrics were used to quantify skull shape, revealing quantitative trait loci (QTL) associated with brachycephaly on CFA1, CFA5, CFA18, CFA30, and CFAX and a missense variant in the bone morphogenetic protein 3 (*BMP3*) gene on CFA32 [8, 9].

A limitation of the aforementioned studies is their disconnected use of phenotype and genotype data. Skulls from osteological collections were used to generate surrogate phenotypes (e.g., ''breed averages'') for use in GWASs [8, 9]. Though this approach has proven successful for detecting QTL, this study design is poorly suited for identifying causal variation, which is not necessarily fixed within breeds whose complex traits are of interest. These breed average study designs cannot utilize mixed-breed dogs that represent a significant portion of extant canines and whose admixture can help separate the phenotypic effects of complex traits. Finally, mapping complex traits, such as canine brachycephaly, is confounded by the need to separate the influences that size has on shape (i.e., allometry) [12].

Our goal was to identify the causal genetic variation responsible for canine brachycephaly. Computed tomography (CT) from 374 dogs that include 84 Kennel Club (UK) recognized breeds and 83 mixed-breed dogs were analyzed using geometric morphometrics. Morphological descriptors, coupled with individuals' genotypes, were used to conduct genome-wide association analyses of skull size and shape. Our analysis of size-controlled skull shape identified a highly significant QTL associated with canine brachycephaly on CFA1, as well as numerous other suggestive associations. Focusing on the CFA1 QTL, we defined a 187.7-kb critical interval common to 30 of 37 brachycephalic dogs. We resequenced 28 brachycephalic dogs to approximately 30-fold depth and filtered polymorphisms within the critical interval against variants called in 319 other resequenced canid genomes. Among five variants that were retained, we detected a long interspersed nuclear element (LINE-1) within the SPARC-related modular calcium binding (*SMOC2*) gene. Transcript analyses revealed alternative splice isoforms that occur in the presence of the LINE-1, causing the incorporation of a premature stop codon after the eighth exon of *SMOC2*'s canonical 13-exon transcript. *SMOC2* mRNA levels are downregulated in a dose-dependent manner with the LINE-1 element. Models of phenotypic effect indicate that the LINE-1 insertion explains up to 36% of facial retrusion observed in our study. Endogenously expressed (mouse) *Smoc2* is observed in the pharyngeal arches during development, and the viscerocrania of *Smoc2*-null mice are dysmorphic. Our data suggest that *SMOC2* dysfunction is responsible for canine brachycephaly. Understanding the developmental role of *SMOC2* could have health implications in human and veterinary medicine.

RESULTS

Canine Phenotypes and GWASs

CTs of referral patients were reconstructed to produce three-dimensional isosurfaces (Figure 1A). We placed 86 landmarks across skull isosurfaces to capture subtle morphological variation within and across patients (Figures 1B–1G and S1). This study included 291 dogs that represented 84 breeds recognized by the Kennel Club (UK). Eighty-three mixed-breed dogs were also included (n = 374; Table S1). Landmarks were analyzed according to morphological substructure (neurocranium, viscerocranium, and mandible; Figure S1). Because form (size and shape considered together) differs so greatly between dogs of various breeds, we performed a Procrustes fit on the landmark data to delineate size, followed by a regression of shape on size to remove the effects of allometry (size-related shape). Principal-component (PC) analysis of distance matrices produced from the regression residuals indicated that the first component, PC1, accounted for 72.2% and 68.8% of variation in the viscerocranium and mandible data, respectively. In the positive direction of viscerocranium PC1, many of the constituent bones of the rostrum narrow mediolaterally and lengthen rostrocaudally. These are shape changes consistent with dolichocephalic dog breeds, such as the smooth collie (Figures 1B and 1F). The opposite phenomena are true for negative PC1: the rostrum broadens and shortens. This reflects the morphological changes that are consistent with brachycephalic head conformation, such as that seen in pugs (Figures 1E and 1F) [8, 13]. Individual breeds cluster together by morphological trait (e.g., viscerocranium shape and neurocranium size; Figure 1G), demonstrating the accuracy of this approach to capture phenotypes and order dogs based on their morphology.

Breeds can also be differentiated from one another by their genomic structure (Figure S2) [9, 14, 15]. Set to k = 2, STRUCTURE revealed the SNP ascertainment bias resulting from the boxer-based dog assembly; breeds closely related to the boxer including the bulldog, Dogue de Bordeaux, and Staffordshire terrier, emerge as a ''molosser'' subpopulation [15]. Approximately one-third of the mixed-breed dogs in our dataset also share this substructure. At k = 84, we observed that the vast majority of owner-reported breed assignments were accurate, though we note evidence of admixture among some of the pedigree dogs.

GWASs of the neurocranium size, as well as viscerocranium and mandible shapes, showed little genetic inflation (Figures 2 and S3). The analysis of neurocranium centroid size identified 32 associated SNPs, representing five genomic loci (Figure 2A; Tables 1 and S2). In a distinction from previous GWASs that investigated body size [16], our data suggest that these loci modulate skull size. This result is particularly surprising for the CFA18 locus, whose underlying *FGF4* retrogene insertion is correlated with limb shortening in breeds like the Dachshund but was not known to reduce skull size, as suggested by our data [17].

Three SNPs on CFA1 at 55862036, 55983871, and 56132332 were associated with viscerocranium PC1 (Figure 2B). GWASs of mandible PC1 also highlighted the CFA1 QTL (Figure S3).

Critical Interval Determination

The CFA1 QTL of viscerocranium and mandible PC1 correspond to a broad selective sweep observed among brachycephalic pedigree dogs [8–11]. Focusing on the CFA1 QTL, we observed 16 SNPs in linkage disequilibrium (LD) (r^2 > 0.2) with the index SNP (BICF2P250912; viscerocranium PC1; p = 1.91 × 10^{-20}; Figure 3A). First, we scanned for haplotype associations

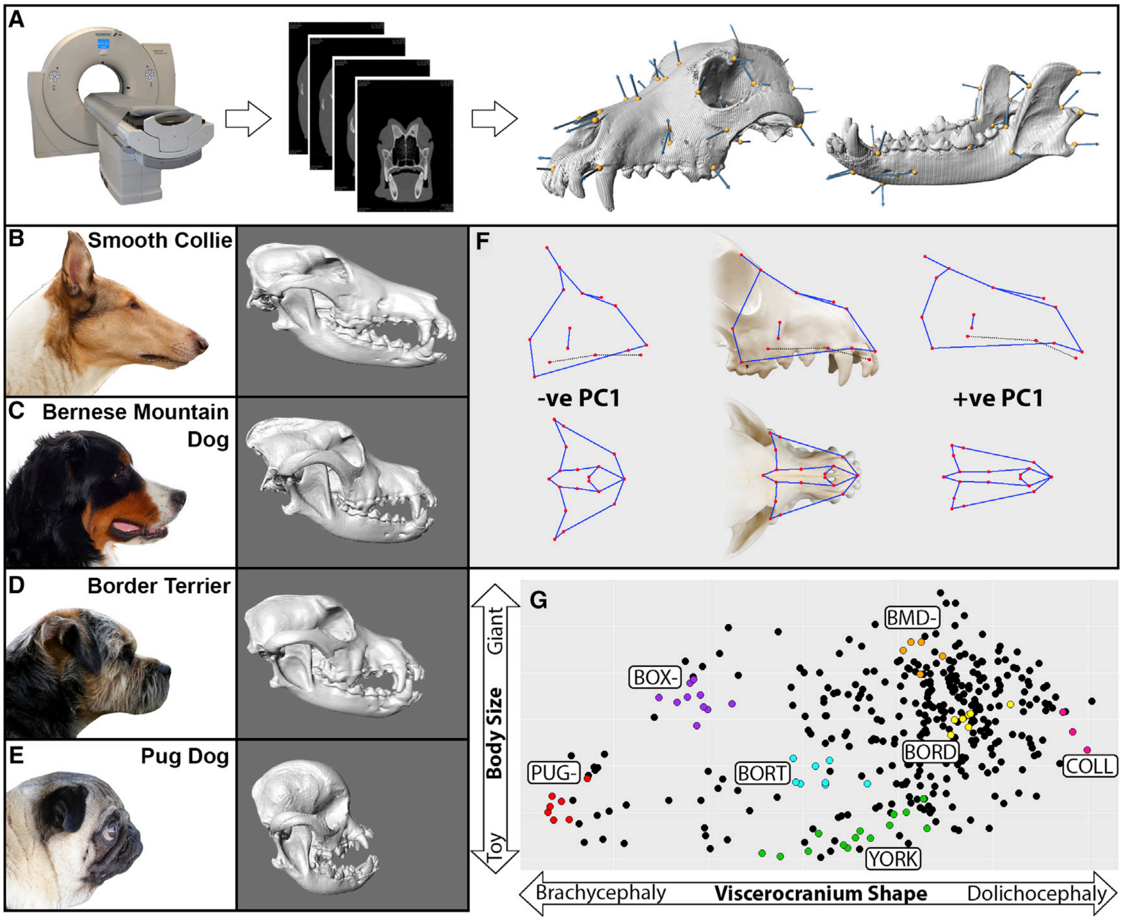

Figure 1. Capturing Gross Interbreed and Subtle Intrabreed Variation in Skull Shape

(A) Three-dimensional isosurfaces of canine skulls are reconstructed from computed tomography (CT) scans of referral patients.

(B–E) Lateral images of a smooth collie (B; dolichocephalic), Bernese mountain dog (C; mesocephalic), border terrier (D; mesocephalic), and pug (E; brachycephalic) with corresponding isosurfaces were included in our analysis. Head images and isosurfaces are not to scale.

(F) Lateral and dorsoventral views of the canine skull with wireframe diagrams superimposed, representing the changes in viscerocranium shape for negative and positive viscerocranium PC1 scores ("ve PC"). Red circles indicate surface landmarks of the rostrum. Connecting blue lines are added to provide visual context to shape. Circles connected by black dotted lines indicate landmarks of the hard palate.

(G) Individual breed members cluster together when viscerocranium shape (viscerocranium PC1) is plotted against body size (neurocranium centroid). BMD-, Bernese mountain dog; BORD, border collie; BORT, border terrier; BOX-, boxer; COLL, smooth collie; PUG-, pug; YORK, Yorkshire terrier.

See also Figures S1 and S2 and Table S1.

extending 1 Mb away from the associated SNPs. This revealed a single region of highly significant haplotypes between 55,881,672 and 56,020,217 (Figure 3B). Genotypes corresponding to this interval, in addition to ~500-kb flanking regions, were phased and ordered in rank of each subject's viscerocranium PC1 value (Figure 3C). As the distribution of viscerocranium PC1 score is bimodal (Figure 2B, inset), with brachycephalic dogs corresponding to PC1 values less than −0.2, we reasoned that the critical interval underlying the CFA1 QTL should be established using haplotypes from this subset of dogs, as constituents are more likely to be fixed for the underlying causal variant(s) (Figure 3C). This revealed a 187.7-kb critical interval (extending between CFA1 55,850,299 and 56,037,676) defined by a 12-SNP haplotype. The 12-SNP haplotype is highly enriched among brachycephalic dogs and was identified among 63 of 74 (85.1%) chromosomes—it is found in just 28 of 674 (4.2%) chromosomes of dogs with viscerocranium PC1 score > −0.2 (Table S3). Suggestive of an effect, the viscerocranium PC1 value of these dogs was significantly different when comparing haplotype carriers to non-carriers (Student's t test; $p = 4.86 \times 10^{-49}$). Curiously, we identified two Dogues de Bordeaux that did not carry the associated haplotype on CFA1. However, our STRUCTURE analysis revealed a higher degree of admixture in these two Dogues de Bordeaux compared to others of the same breed (Figure S2), suggesting that they were cryptic outbreds. Moreover, both dogs had longer viscerocrania than those Dogues de Bordeaux that were fixed for the 12-SNP haplotype (data not shown).

Eight of the twelve SNPs of this haplotype are located within the SPARC-related modular calcium-binding protein 2 (*SMOC2*) gene (Figure 3C). The remaining four SNPs are spread across ~43 kb of sequence downstream of the gene.

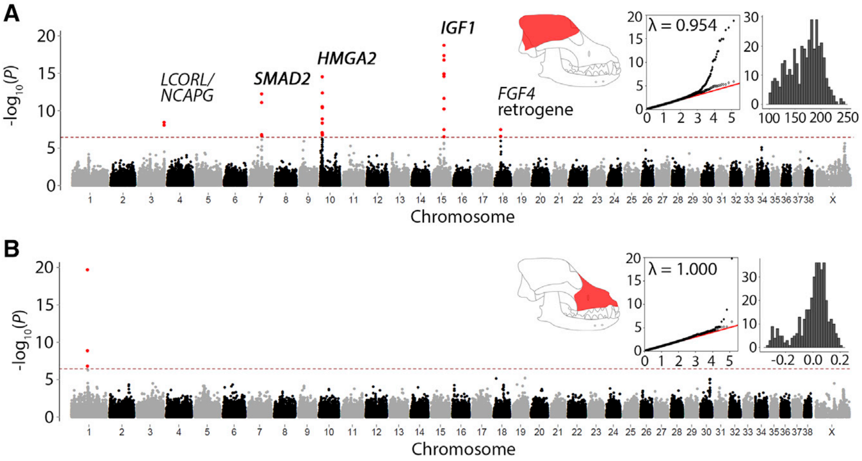

Figure 2. Morphology of Skull Substructures Are Associated with Multiple QTL

Manhattan plots for neurocranium centroid size (A) and viscerocranium PC1 GWASs (B). Red dashed line (3.6×10^{-7}) indicates threshold for multiple testing with significant SNPs colored red. The associated SNPs and candidate genes at each locus are summarized in Table 1. Insets: skull schematics indicate the region of landmarks used for datasets. Expected (x axis) and observed (y axis) $-\log^{10}$(p) values are plotted for all SNPs (black circles) and pruned SNPs (gray circles). Histograms depict the frequency (y axis) of viscerocranium PC1 and neurocranium centroid, respectively. See also Figures S1 and S3 and Table S2.

Variant Filtering Analysis

Focusing on the CFA1 critical interval, we analyzed 187,377 bp of whole-genome sequence. In total, we called 3,674 SNPs/INDELS and 162 structural variants (Table 2). After hard filtering (Table S4), four SNPs and one structural variant remained as candidates for further consideration (Table 2; see STAR Methods). All five remaining variants are located within introns of the *SMOC2* gene. The structural variant is a 1,531-bp insertion, which is present in the dog reference genome (which was generated from a brachycephalic breed—a boxer). The SNPs and insertion appear in complete linkage disequilibrium (data not shown). Though we cannot formally exclude their contribution to brachycephaly, none of the SNPs fell in regions of high conservation across species (Figure S4). Thus, their potential to cause brachycephaly was poorly supported.

Conversely, the insertion is a 3′ truncated fragment of a class 1 long interspersed nuclear element (LINE-1). LINE-1 insertions are known to be mutagenic in both man and dogs [18, 19]. The LINE-1 insertion within *SMOC2* is fragmented, possibly due to incomplete insertion through "abortive" retrotransposition, and includes an intact 3′ UTR and 1,302 bp of ORF2 (Figure 4A) [20]. We genotyped the LINE-1 fragment in subjects used in our GWASs. The LINE-1 fragment is found among 91.5% of chromosomes of brachycephalic dogs (viscerocranium PC1 < −0.2) compared to only 2.1% of chromosomes of non-brachycephalic dogs (Figure 4B). The LINE-1 fragment appears to have no correlation with neurocranium centroid size (Figure 4C). Grouping individuals based on the number of LINE-1 fragment alleles they carried, we observe an additive effect for all normalized linear measurements taken from skull isosurfaces, with the greatest effect observed on the length of the palatine bone (Figures 4D and 4E).

LINE-1 retrotransposons are known to alter local gene expression through a variety of mechanisms that affect transcription [21–23]. Therefore, we quantified the relative expression levels of *SMOC2* mRNA at both the 3′ and 5′ ends of the transcript. A comparable additive effect on *SMOC2* expression was observed across the transcript (Figure 4F). Subjects that were homozygous for the *SMOC2* LINE-1 allele had an ~5-fold reduction in total *SMOC2* mRNA expression compared to individuals without a copy of the allele. This observation was independently confirmed by RNA sequencing. Subjects that were homozygous carriers for the LINE-1 allele similarly had a significant reduction in total *SMOC2* mRNA levels when compared to non-carriers (fold change = 3.1; Figure 4G). Three additional genes showed significantly reduced expression, including two novel genes for long non-coding RNAs (ENSCAFG00000039143 and ENSCAFG00000035778) and the protein-coding urotensin 2B (*UTS2B*) gene. None of these genes are located on CFA1. No changes in expression of the neighboring genes to *SMOC2*, *THBS2*, and *DACT2* (dishevelled-binding antagonist of beta-catenin 2) were observed (Figure 4G). Non-carriers of the *SMOC2* LINE-1 exclusively transcribed the "canonical" 13-exon transcript of *SMOC2* (Figure 4H). Homozygous carriers

Table 1. SNPs Showing Genome-wide Significance with Skull Datasets

Dataset[a]	Chromosome	Index SNP	Position	Candidate Gene	Allele[b]	p Value
Viscerocranium	1	BICF2P250912	55,983,871	*SMOC2**	G > A	1.91×10^{-20}
Mandible	1	BICF2P250912	55,983,871	*SMOC2**	G > A	8.43×10^{-10}
Neurocranium	3	TIGRP2P56799_rs8666557	91,103,945	*LCORL/NCAPG*	G > T	3.64×10^{-9}
Neurocranium	7	BICF2S23352941	43,719,549	*SMAD2**	A > G	5.71×10^{-13}
Neurocranium	10	G580f46S240	8,183,593	*HMGA2*	C > T	3.06×10^{-15}
Neurocranium	15	BICF2P355320	41,257,020	*IGF1**	C > T	1.73×10^{-19}
Neurocranium	18	BICF2S23615757	20,272,961	*FGF4* retrogene	A > C	3.31×10^{-8}

See also Table S2. For intragenic SNPs, genes are denoted by asterisks.
[a]Only index SNPs are listed. A complete list of significant SNPs is shown in Table S2.
[b]Derived alleles are shown after ancestral alleles

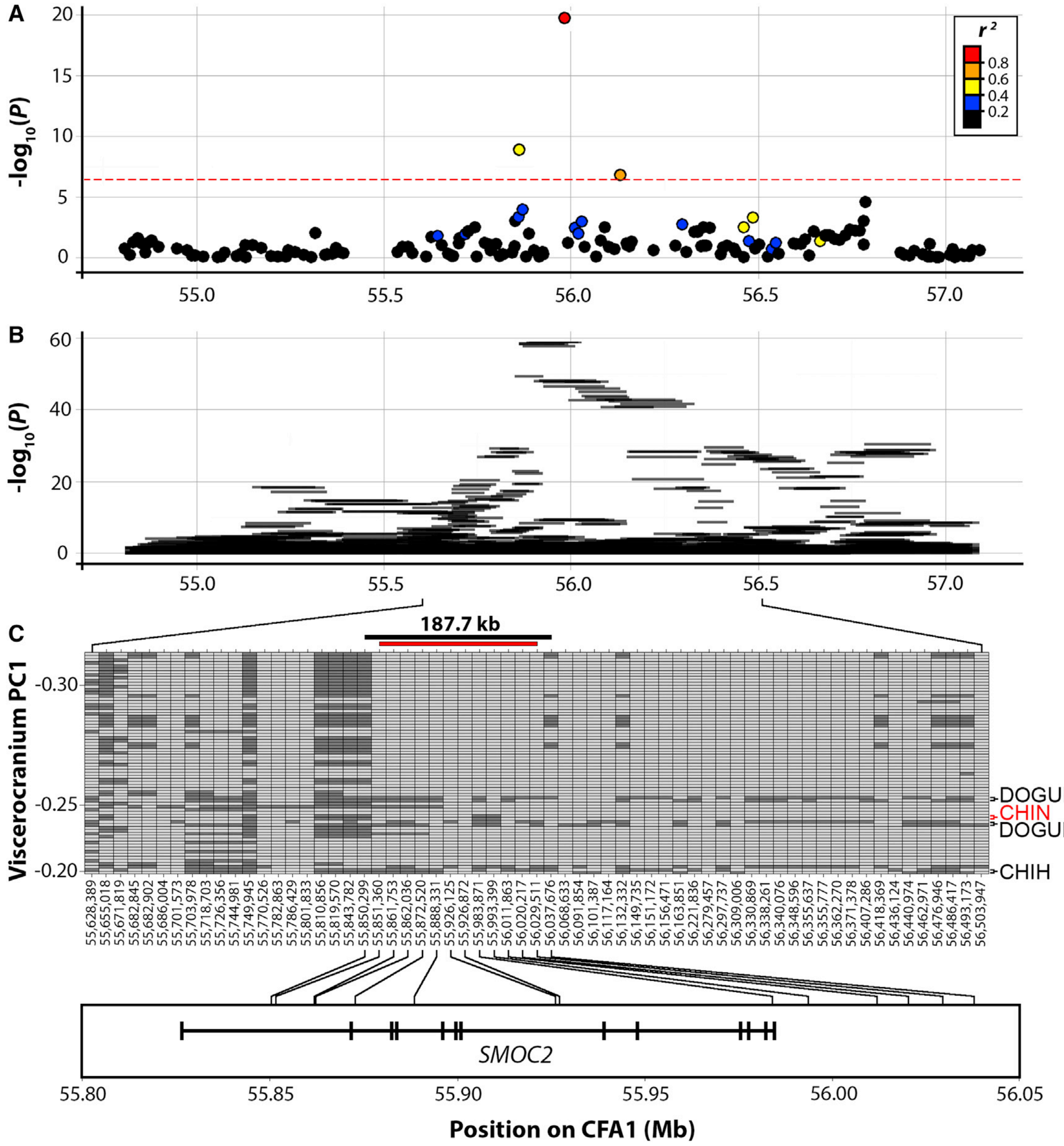

Figure 3. Regional Association and Critical Interval Determination of the CFA1 Viscerocranium QTL

(A) SNP associations with viscerocranium PC1 are shown for ~1 Mb on either side of significant SNPs on CFA1. SNPs are colored depending on the degree of LD (r^2) with the index SNP (BICF2P250912; 1.91×10^{-20}).

(B) Ten-SNP sliding window haplotype association.

(C) Genotypes between 55,881,672 and 56,020,217 (including ~500 kb of flanking sequence) were phased and ranked by their viscerocranium PC1 value. Only haplotypes from brachycephalic dogs (viscerocranium PC1 ≤ −0.2; see Figure S3) were considered. Haplotypes are paired by subject and ranked by viscerocranium PC1 value. Alleles colored light gray match the consensus haplotype; dark gray alleles are variant. A 187.7-kb critical interval is defined by at least three meiotic recombinations (indicated above by black bar). The 12 SNPs that constitute the associated haplotype (red bar) are distributed within or up to ~44 kb downstream of *SMOC2*. Black arrows indicate 3 of 37 dogs that have a homozygous variant haplotype. These dogs are registered as two Dogues de Bordeaux and a Chihuahua. The red arrow indicates a Japanese Chin that is homozygosed for a recombinant haplotype within the critical interval.

See also Figures S2 and S6 and Table S3.

for the *SMOC2* LINE-1 similarly transcribed the canonical transcript; however, in addition to this, these individuals also transcribed multiple different isoforms of *SMOC2* (Figure 4H). Using primers designed against exon 8 and the LINE, we identified three isoforms present across all individuals homozygous for the LINE-1 element and a further three rarer isoforms present in homozygous or heterozygous carriers of the LINE-1 element (Figure S5; Table S5). All isoforms incorporate the LINE-1 element and differing lengths of preceding intron into the *SMOC2* mRNA following exon 8. Each of the different splice sites within intron 8 are preceded by an adenine and guanine residue (AG)—an almost invariant characteristic of mammalian splice

Table 2. Variant Filtering within the Viscerocranium Critical Interval

Software	GATK/SnpSft	Pindel
Variant type	SNPs/INDELS	Structural variants
Base pairs analyzed	187,377	187,377
Pre-filtering	3,674	162
Post-filtering	4	1

Filtering criteria are listed in Table S4. See also Figure S4 and Table S4.

acceptors (Table S5) [24, 25]. All alternative isoforms are predicted to introduce a premature stop codon following exon 8. It is unclear whether the alternative truncated isoforms are translated; however, we predict the protein products would shear within the thyroglobulin-like domain and would have no extracellular calcium-binding domain (Figure 4I) [26].

In exon 8, we observed a SNP that encodes a silent C/T substitution at position 55,939,143. Interestingly, both the C and T alleles are present across "ancestral" populations that do not carry the LINE-1 element. However, the LINE-1 element is only observed in the presence of exon 8's T allele (Figure 4H). This suggests that the C/T variant predates the insertion of the LINE-1 variant. In heterozygous subjects, the C/T variant enabled us to quantify the allele-specific transcriptional activity of *SMOC2*. Transcripts from a Yorkshire terrier dog that was homozygous ancestral for the *SMOC2* allele (lacking the LINE-1), but heterozygous for the C/T allele, had an allele percentile ratio of 46:54, suggesting that transcripts from both alleles are equally represented (Table S6). In contrast, two Cavalier King Charles spaniels that were heterozygous for both the *SMOC2* LINE-1 and the C/T allele had allele percentile ratios of ~75:25, which indicates that the DNA allele with the LINE-1 element contributes fewer of the *SMOC2* reads (Table S5). A lower abundance of transcripts incorporating the LINE-1 element may suggest that they are targeted by nonsense-mediated decay, decreased transcriptional activity, or both.

Size-Effect Modeling on Skeletal Size and Shape

We were interested in modeling phenotypic effects of size and shape using the skull-derived QTL described by our study and elsewhere [8, 9, 16, 27]. The derived allele frequencies of associated markers of *SMOC2*, CFA30 QTL, *BMP3*, *IGF1*, and *STC2* differ significantly according to viscerocranium PC1 (Figure 5A). These five genotypes were applied as explanatory variables in a linear stepwise model for the viscerocranium PC1. Alone, the homozygous-derived alleles of the *SMOC2* LINE-1 explain the largest amount of viscerocranium variation (R^2 = 36%), with markers at the CFA30 QTL, *BMP3 IGF1*, and *STC2* explaining 28%, 12%, 4%, and 4%, respectively (Figure 5B). These variances are not additive but infer the maximum potential contribution of each genotype. Together, 45% of viscerocranium's proportion of variation explained (PVE) is explained by these five genotypes. *IGF1*, *IGF1R*, *SMAD2*, *FGF4*, *GHR*(1), *GHR*(2), CFA30 QTL, *BMP3*, *STC2*, *HMGA2*, and the *LCORL/NCAPG* locus are significantly associated with neurocranium centroid size (Figure 5C). The best model for explaining variation in neurocranium centroid size selected a subset of genotypes (*SMAD2*, IGF1, *FGF4*, *IGF1R*, and the *LCORL/NCAPG* locus), which together explain up to 68% PVE (Figure 5D). Individually, the homozygous-derived alleles of *SMAD2*, *HMGA2*, *GHR*(1), *IGF1*, *FGF4*, *STC2*, *IGF1R*, the *LCORL/NCAPG* locus, *GHR*(2), the CFA30 locus QTL, and *BMP3* explain up to 47%, 37%, 31%, 29%, 28%, 22%, 21%, 14%, 10%, 8%, and 6% of neurocranium centroid size variation, respectively (Figure 5D).

Species Conservation of *SMOC2*

Morpholino knockdown of zebrafish *smoc2* suggests it regulates head development [28, 29]. To determine whether *SMOC2* function is evolutionary conserved across other species, we first assessed its regional conservation by aligning the locus to the human genome. Mouse and chick sequence conservation was strikingly reduced compared to other species, including the dog (Figure S6A). Despite this, embryonic expression in chick and mice is observed in the first pharyngeal arch (Figure S6) [30]. Notably, the cranial neural crest streams into the first arch to populate the primordia that will give rise to the maxilla, as well as other constituents of the viscerocranium and mandible [31–33]. Previous to our study, $Smoc2^{-/-}$ mice were generated and phenotyped for the International Mouse Phenotyping Consortium (IMPC). Although these mice are no longer maintained, adults used for phenotyping were viable and fertile. We assessed archived radiographs of $Smoc2^{-/-}$ (n = 8) and strain-specific controls (n = 4; Figure S7A). Principal-component analysis of the whole head revealed similar morphological variation to that which we observed in dogs. Murine PC1 variation showed mediolateral widening and rostrocaudal shortening of the skull (Figure S7B). PC1 values clustered differentially by genotype (*Smoc2* knockout versus control; $p < 0.001$; Figure S7C); however, no such segregation was observed for sex (Figure S7D). Total palate length was assessed from lateral radiographs. The palate was significantly shorter in transgenic mice (Student's t test; $p = 0.0011$), though not when allometry was removed (Figure S7E; data not shown). Given this observation and the fact that the locus is poorly conserved might suggest species-level differences in *Smoc2* function. Nonetheless, our mouse data, as well as additional bone phenotypes described by the IMPC, indicate that disruption of *Smoc2* is sufficient to adversely affect craniofacial biology.

DISCUSSION

Studies, including ours, continue to demonstrate the effectiveness of dog breeders at propagating aesthetic traits [8, 17, 34]. This cultivation of morphologies predated the formation of breed clubs. The selective sweep and association of the CFA1 QTL with brachycephaly was recognized in the early days of dog GWASs; however, confirmation of the underlying causative genetics remained elusive. Unlike QTL mapping, fine mapping approaches based on haplotype comparisons are confounded by the occasional "outlier" within a breed that is not fixed for or does not even carry the genetic variant that drives a trait that is common to other members of its breed. Moreover, whereas dog traits (e.g., brachycephaly) that are common across subsets of breeds are often driven by identity-by-descent genetics, this phenomenon is not absolute. To avoid these issues, as well as leverage the genetics of mixed-breed dogs, we built a study population whose phenotypes and genotypes were derived individually.

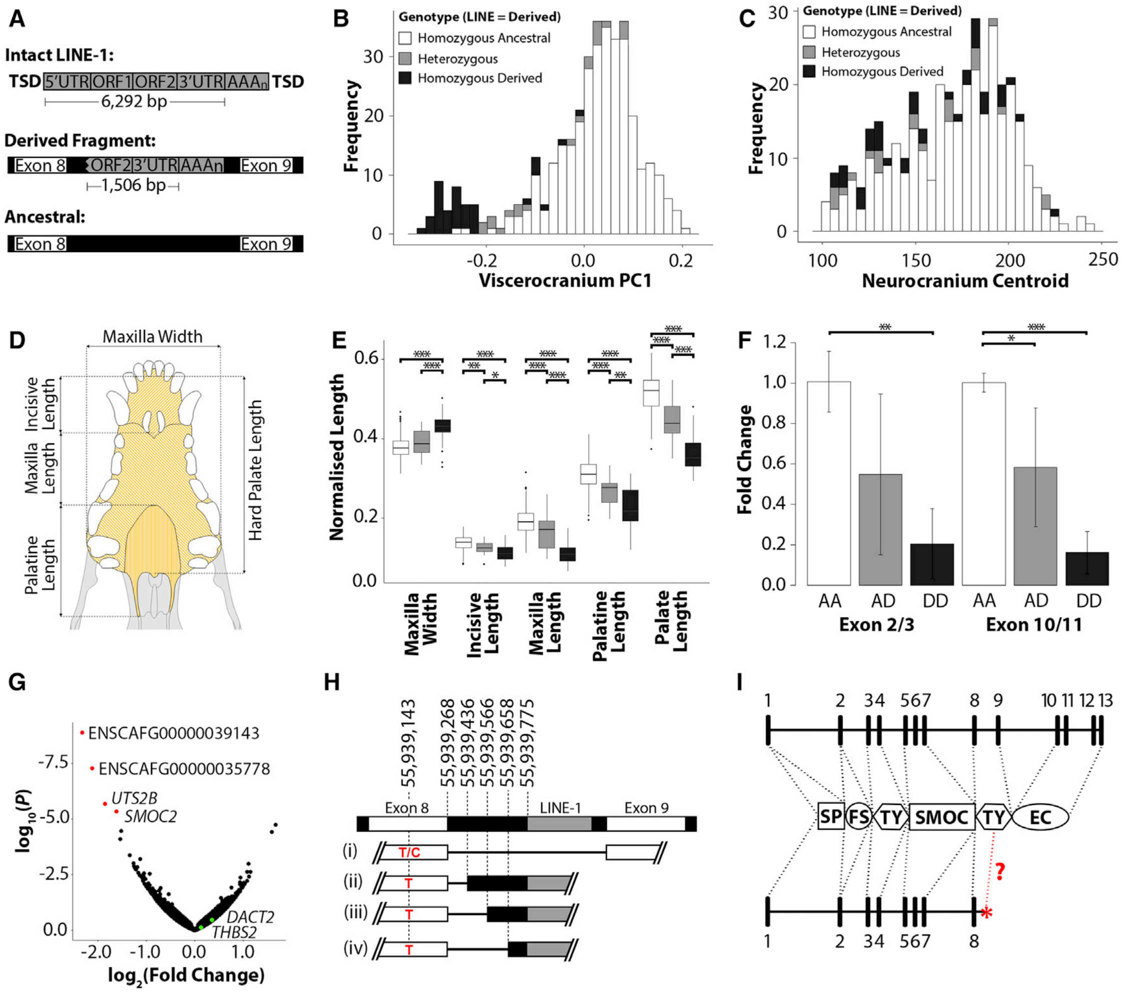

Figure 4. Characterization of the Intronic LINE-1 Retrotransposon within *SMOC2*

(A) Schematic of a full-length canine LINE-1 element consisting of 5′ UTR/3′ UTR, open reading frames 1 and 2 (ORF1/ORF2), and a polyadenylated tail ($AAAA_n$) flanked by target site duplications (TSD). The structural variant within *SMOC2* is 1,506 bp in length (in addition to a poly(A) tail) and has a 99.1% match to the consensus sequence of canine LINE-1.

(B and C) Distribution of the *SMOC2* LINE-1 fragment for (B) viscerocranium PC1 and neurocranium centroid size (C) across all individuals.

(D) Ventral-dorsal view of the canine hard palate and its constituent bones.

(E) Length and width of the canine palate and constituent bones normalized by the neurocranium centroid for homozygous ancestral (white), heterozygotes (gray), and homozygous-derived (black) individuals for the *SMOC2* LINE-1 insertion.

(F) Relative expression levels of *SMOC2* both up- and downstream of the LINE insertion (<0.05 *; <0.01 **; <0.001 ***). Error bars represent SEM.

(G) RNA sequencing (RNA-seq) data reveal four genes with significant changes in mRNA levels (red) for homozygous *SMOC2* LINE-1 carriers compared to non-carriers (three each). Neighboring genes to *SMOC2* are colored green.

(H) Schematic of genomic DNA (gDNA) spanning exon 8 and 9 of *SMOC2*, including the LINE-1 fragment. mRNA transcripts include the canonical splicing of *SMOC2* (i) followed by the three most abundant *SMOC2* isoforms when the LINE-1 element is present (ii–iv). All isoforms have premature stop codons prior to exon 9. C/T indicates the SNP in exon 8. Schematic is not to scale.

(I) Exons 1–13 of *SMOC2* contribute to a follistatin-like module (FS), thyroglobulin-like modules (TY), a unique SMOC module, an extracellular calcium-binding module (EC), and a signal peptide (SP).

See also Figures S5 and S7 and Tables S5 and S6.

We distilled the CFA1 locus to reveal a haplotype overlapping with *SMOC2* as the major contributor to brachycephaly. We strongly suspect that the insertion of a truncated transposable element into *SMOC2* is most likely causal; however, we acknowledge the limitations of our study. The dog's long-range linkage disequilibrium prevented us from disassociating four SNPs that are in linkage disequilibrium with the LINE. Whether or not these variants have functional impacts cannot be dismissed. Second, whereas our transcriptional analysis demonstrates differential expression and missplicing of *SMOC2* that are associated with the LINE insertion, we cannot say whether other genes are affected by this haplotype in *cis*. Due to limited tissue availability, we restricted our differential expression to testis, a tissue where *SMOC2* was assumed to be highly expressed based on evidence from other species [35, 36]. In the future, additional tissues will need to be tested to determine whether genes in *cis*

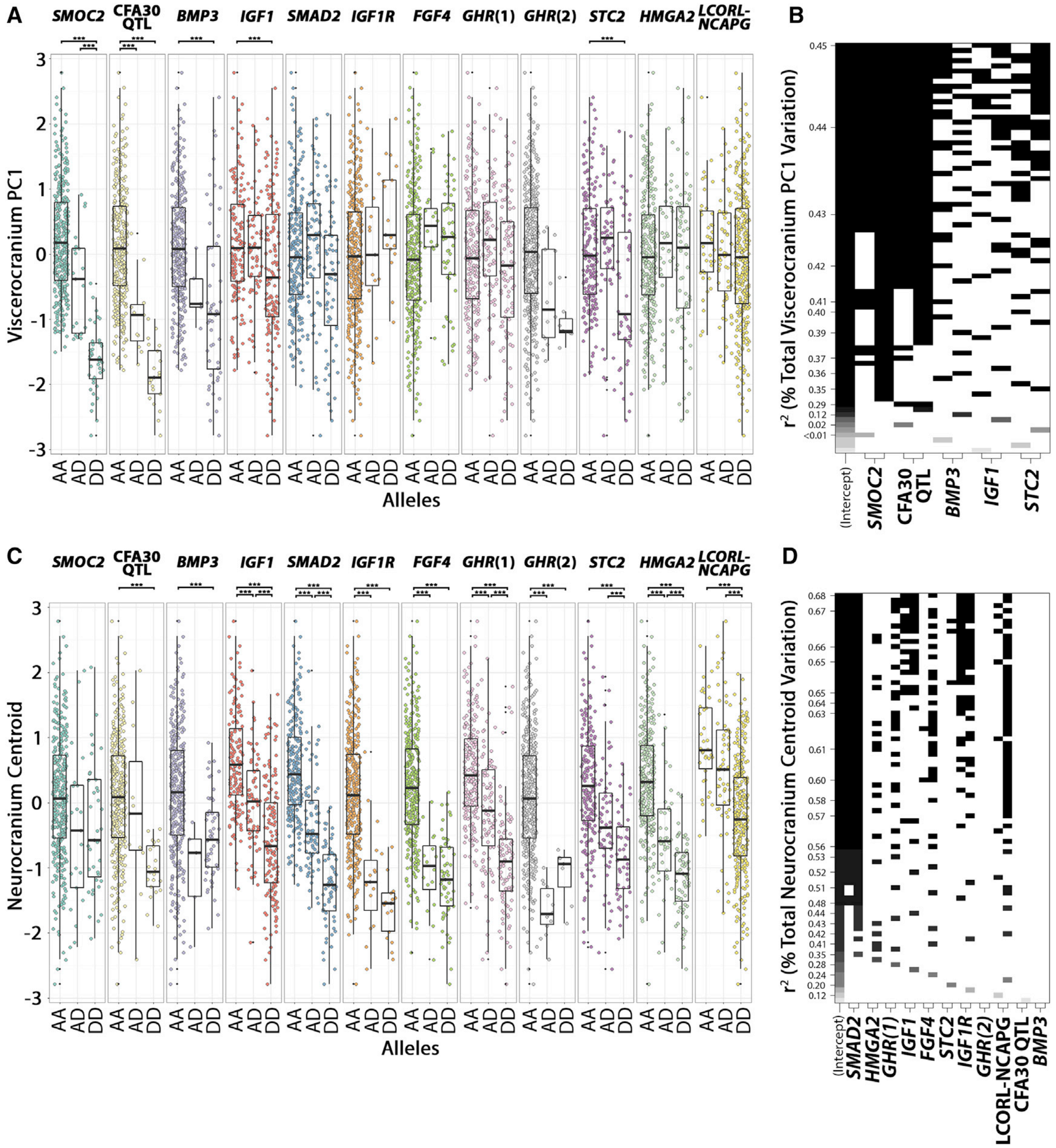

Figure 5. Size Effects of the Viscerocranium Shape and Neurocranium Centroid Size QTL

(A and C) Boxplots depicting the distribution of normalized size-corrected viscerocranium PC1 (A) and normalized neurocranium centroid size (C) for 11 loci linked with body size and skull shape. Distributions are subdivided by genotype —homozygous ancestral (AA), heterozygotes (AD), and homozygous derived (DD). *** denotes $p < 0.001$ in Mann-Whitney-Wilcoxon and Kolmogorov-Smirnov tests.

(B and D) A stepwise linear regression model for viscerocranium PC1 (B) and neurocranium centroid (D) determined the best explanatory model for ancestral (left) and derived (right) genotypes for each positional candidate.

are differentially expressed in association with the haplotype we describe.

Modeling phenotypic variance was enhanced by the inclusion of mixed-breed dogs, whose admixed genomes and lack of standardization helped separate QTL that would otherwise co-segregate. Alone, *SMOC2* explains up to 38% of viscerocranium PC1 variance. Whereas clearly the locus has a large effect size, our study is currently underpowered to exhaustively detect QTL that modulate brachycephaly or, more broadly, shape of the facial skeleton. This is underscored by the fact that we have not explained canine brachycephaly as it occurs in two Dogues de Bordeaux and an Affenpinscher (the latter was used in our whole-genome sequencing); none showed evidence of a selective sweep on CFA1 nor did they carry the associated 12-SNP haplotype. Moreover, our GWASs failed to replicate the CFA32/*BMP3* and CFA30 QTL associations described previously [8]. A likely explanation for this is the modest numbers of small, brachycephalic breeds in our study, as well differing

demographics. Our study is lacking in Brussel Griffon, Pekingese, Boston terriers, and Japanese Chin—all brachycephalic breeds whose members are typically homozygous for the missense variant in *BMP3*.

By necessity, we cannot explain the genetics of skull shape without addressing confounding effects of allometry, which is essential in a species whose size differential can exceed 40-fold. We used subjects' neurocranium centroid size to remove the influences of allometry from viscerocranium shape variation, as well as to explore the genetics of neurocranium size itself. A genomic association of neurocranium centroid size identified five loci. Four of these loci were previously identified in body size studies across a variety of species: SMAD family member 2 (*SMAD2*) [9, 16, 27], high-mobility group AT-hook 2 (*HMGA2*) [9, 16, 27, 37–39], insulin-like growth factor 1 (*IGF1*) [16, 27], and the ligand-dependent nuclear receptor corepressor-like (*LCORL*)/non-SMC condensin I complex, subunit G (*NCAPG*) locus [16, 40–42]. Our effect size data point to their relative contribution to neurocranium centroid size; the largest effect size is explained by the putative enhancer deletion at the *SMAD2* locus [27]. The association of neurocranium centroid size with the fibroblast growth factor 4 (*FGF4*) retrogene was unexpected. Parker et al. [17] first identified an *FGF4* retrogene associated with canine asymmetric chondrodysplasia, a form of dwarfism that gives breeds like the Dachshund its short legs. The same locus was associated with body weight [16], though this could be explained by reduced leg mass. Our results indicate that the bone-based structure of the neurocranium is also reduced in size by the retrogene. Similarly, Hayward et al. [16] identified an association to stature and body weight in proximity to *SMOC2* [16]. Because a high proportion of the brachycephalic dog population are low-to-medium weight breeds (Figure 1G), the interpretation of their association is unclear. In our study, we see no evidence that the *SMOC2* locus modulates neurocranium centroid size (Figures 2A and 5C) and, by extension, skeletal size. However, we cannot exclude the possibility that the QTL noted by the authors affect soft tissue mass or appendicular bone length.

SMOC2 belongs to the BM-40 (SPARC) family of matricellular proteins, which contain an extracellular calcium-binding module and a follistatin-like domain. *SMOC2* is distinguished from the BM-40 family by the addition of two thyroglobulin domains and a novel domain unique to the SMOC subgroup [26]. The calcium-binding module facilitates the binding of multiple collagen types [43] and the interaction with several growth factors [44, 45], which permits the proteins to function in cell adhesion, cell proliferation, and matrix turnover (reviewed by [46]). The BM-40 family was first identified in bone (where *SMOC2* has been shown to be differentially expressed across the growth plate) but has since been found in a wide variety of other tissues [38, 47–49]. Mounting evidence suggests the *SMOC2* plays an important role in craniofacial form across species. Knockdown of zebrafish *smoc2* causes severe craniofacial hypoplasia [28], a process that may act by downregulating target genes of bone morphogenetic protein (BMP) signaling [50]. In chick embryos, *Smoc2* is prominently expressed in the pharyngeal arches. Murine craniofacial development undergoes dynamic growth between embryonic days 10.5 and 12.5. Throughout this window, *Smoc2* is shown to have differential temporal expression in the frontonasal process and maxillary/mandibular prominences [47]—tissues that give rise to mandible and viscerocranial structures. Our geometric morphometric analysis of radiographs indicate the skulls of *Smoc2*-null mice cluster distinctly from wild-type, though a detailed understanding of the shape changes that occur in null mice will require three-dimensional analysis (Figure S7). It is intriguing that numerous copy-number variants spanning *SMOC2* are associated with human phenotypes, including brachycephaly, hydrocephalus, long face (vertical), and hypertelorism [51]. Point mutations in *SMOC2* were identified independently in patients with dentin dysplasia type I syndrome, whose hallmarks include severe oligodontia and microdontia [29, 52]. Finally, deleterious mutations in *SMOC2* were identified in DECODE [53] and Generation Scotland biobanks (M.L.B., unpublished data).

Leveraging the craniofacial diversity of dogs, we set out to discover candidate genes involved in human craniofacial anomalies, particularly craniosynostosis and midface hypoplasia. Our results suggest that *SMOC2* should be screened as a candidate for diagnosis. Not to be ignored, the role of *SMOC2* dysfunction and the associated haplotype we defined need further exploration as they concern the health of brachycephalic dogs. As our canine skull project continues to grow, we will explore the role of *SMOC2* and other skeletal QTL with comparative health implications.

STAR★METHODS

Detailed methods are provided in the online version of this paper and include the following:

- KEY RESOURCES TABLE
- CONTACT FOR REAGENT AND RESOURCE SHARING
- EXPERIMENTAL MODEL AND SUBJECT DETAILS
- METHOD DETAILS
  - DNA Extraction and Microarray Genotyping
  - RNA Extraction and qPCR
  - Sequencing Library Preparations
  - Histology
- QUANTIFICATION AND STATISTICS
  - Morphometrics
  - Genotype Analyses
  - Fine Mapping
  - Variant Filtering
  - qRT-PCR
  - RNA-Seq
  - Generation Scotland: Scottish Family Health Study
- DATA AND SOFTWARE AVAILABILITY
- ADDITIONAL RESOURCES
  - DECIPHER

SUPPLEMENTAL INFORMATION

Supplemental Information includes seven figures and six tables and can be found with this article online at http://dx.doi.org/10.1016/j.cub.2017.04.057.

AUTHOR CONTRIBUTIONS

Conceived and designed the experiments, T.W.M., E.J.J., R.J.M., D.N.C., and J.J.S.; performed the experiments, T.W.M., E.J.J., L.M., and J.J.S.; performed

the data analysis, T.W.M., E.J.J., M.L.B., L.M., M.G.D., and J.J.S.; diagnostic image collection, C.I.J., A.G., T. Liuti, S.G., J.L., D.J.A., R.J.M., and T.S.; biobanking, T.W.M., E.J.J., C.I.J., A.G., M.N., D.K., M.D., R.M.P., D.J.A., G.T.H., T. Leeb, R.J.M., and J.J.S.; mouse data, K.S.; wrote the manuscript, T.W.M., G.T.H., and J.J.S. All authors revised the manuscript.

ACKNOWLEDGMENTS

The authors thank the many dog owners whose beloved pets enabled this study. We would also like to acknowledge the many clinicians at the R(D)SVS Hospital for Small Animals, PTDS, Dick White Referrals, Kleintierklinik of Vetsuisse Faculty Bern, and Friendship Animal Hospital, who helped us collect biospecimens used in our DNA and RNA analysis. Gonad tissue was collected by J.J.S. with the support of E. Ostrander. Special recognition is given to D. Gaylor, S. Campbell, J. Lawrence-Rausch, R. Elders, K. Jermyn, A. de Castro Marques, S. Woods, Y. Martinez-Pereira, B. Corcoran, T. Nuttall, D. Gow, K. Mellanby, and N. Bommer for their extraordinary efforts to enable patient recruitment; the Dog Biomedical Variant Database Consortium (G. Aguirre, C. André, D. Bannasch, D. Becker, C. Drögemüller, O. Forman, S. Friedenberg, E. Furrow, U. Giger, C. Hitte, M. Hytönen, H. Lohi, C. Mellersh, J. Mickelson, A. Oberbauer, S. Schmutz, and C. Wade) for sharing whole-genome sequencing data from control dogs; N. Russel for providing skull photography; and C. Muller, K. McLellan, M. John, and K. Thomas for providing animal photography. Additional thanks are given to G. Faulkner, D. FitzPatrick, I. Jackson, M. McGrew, H. Sang, A. Balic, and R. Harrington for their helpful suggestions. The mouse *Smoc2* riboprobe was generously provided by J. Rainger. J.J.S. is a University of Edinburgh Chancellor's fellow and received funding from the Wellcome Trust-University of Edinburgh Institutional Strategic Support Fund (ISSF2). The Roslin Institute receives strategic funding from the Biotechnology and Biosciences Research Council: J.J.S. (BB/J004235/1 and BB/P013759/1) and M.D. (BB/J004316/1 and BB/P013732/1). J.J.S. and M.N. received funding from the Albert Heim Foundation (grant 101 13.03.2012). T. Leeb received funding from the Albert Heim Foundation (grant 105 09.10.2012). Funding for DECIPHER was provided by the Wellcome Trust.

Received: October 19, 2016
Revised: March 14, 2017
Accepted: April 27, 2017
Published: May 25, 2017

REFERENCES

1. Stockard, C.R. (1941). The Genetic and Endocrinic Basis for Differences in Form and Behaviour (Philadelphia: The Wistar Institute of Anatomy and Biology).
2. Wayne, R.R. (2001). Phylogeny and origin of the domestic dog. In The Genetics of the Dog, A. Ruvinsky, and J. Sampson, eds. (CABI), pp. 1–14.
3. Harvey, R.G., and ter Haar, G. (2016). Brachycephalic obstructive airway syndrome. In Ear, Nose and Throat Diseases of the Dog and Cat, R.G. Harvey, and G. ter Haar, eds. (Devon: CRC Press), pp. 290–293.
4. Poncet, C.M., Dupre, G.P., Freiche, V.G., Estrada, M.M., Poubanne, Y.A., and Bouvy, B.M. (2005). Prevalence of gastrointestinal tract lesions in 73 brachycephalic dogs with upper respiratory syndrome. J. Small Anim. Pract. *46*, 273–279.
5. Sanchez, R.F., Innocent, G., Mould, J., and Billson, F.M. (2007). Canine keratoconjunctivitis sicca: disease trends in a review of 229 cases. J. Small Anim. Pract. *48*, 211–217.
6. Collmann, H., Sörensen, N., and Krauss, J. (2005). Hydrocephalus in craniosynostosis: a review. Childs Nerv. Syst. *21*, 902–912.
7. Wilkie, A.O.M., Byren, J.C., Hurst, J.A., Jayamohan, J., Johnson, D., Knight, S.J.L., Lester, T., Richards, P.G., Twigg, S.R.F., and Wall, S.A. (2010). Prevalence and complications of single-gene and chromosomal disorders in craniosynostosis. Pediatrics *126*, e391–e400.
8. Schoenebeck, J.J., Hutchinson, S.A., Byers, A., Beale, H.C., Carrington, B., Faden, D.L., Rimbault, M., Decker, B., Kidd, J.M., Sood, R., et al. (2012). Variation of BMP3 contributes to dog breed skull diversity. PLoS Genet. *8*, e1002849.
9. Boyko, A.R., Quignon, P., Li, L., Schoenebeck, J.J., Degenhardt, J.D., Lohmueller, K.E., Zhao, K., Brisbin, A., Parker, H.G., vonHoldt, B.M., et al. (2010). A simple genetic architecture underlies morphological variation in dogs. PLoS Biol. *8*, e1000451.
10. Quilez, J., Short, A.D., Martínez, V., Kennedy, L.J., Ollier, W., Sanchez, A., Altet, L., and Francino, O. (2011). A selective sweep of >8 Mb on chromosome 26 in the boxer genome. BMC Genomics *12*, 339.
11. Bannasch, D., Young, A., Myers, J., Truvé, K., Dickinson, P., Gregg, J., Davis, R., Bongcam-Rudloff, E., Webster, M.T., Lindblad-Toh, K., and Pedersen, N. (2010). Localization of canine brachycephaly using an across breed mapping approach. PLoS ONE *5*, e9632.
12. Wayne, R.K. (1986). Cranial morphology of domestic and wild canids: the influence of development on morphological change. Evolution *40*, 243–261.
13. Drake, A.G., and Klingenberg, C.P. (2010). Large-scale diversification of skull shape in domestic dogs: disparity and modularity. Am. Nat. *175*, 289–301.
14. Pritchard, J.K., Stephens, M., and Donnelly, P. (2000). Inference of population structure using multilocus genotype data. Genetics *155*, 945–959.
15. Parker, H.G., Kim, L.V., Sutter, N.B., Carlson, S., Lorentzen, T.D., Malek, T.B., Johnson, G.S., DeFrance, H.B., Ostrander, E.A., and Kruglyak, L. (2004). Genetic structure of the purebred domestic dog. Science *304*, 1160–1164.
16. Hayward, J.J., Castelhano, M.G., Oliveira, K.C., Corey, E., Balkman, C., Baxter, T.L., Casal, M.L., Center, S.A., Fang, M., Garrison, S.J., et al. (2016). Complex disease and phenotype mapping in the domestic dog. Nat. Commun. *7*, 10460.
17. Parker, H.G., VonHoldt, B.M., Quignon, P., Margulies, E.H., Shao, S., Mosher, D.S., Spady, T.C., Elkahloun, A., Cargill, M., Jones, P.G., et al. (2009). An expressed fgf4 retrogene is associated with breed-defining chondrodysplasia in domestic dogs. Science *325*, 995–998.
18. Choi, Y., Ishiguro, N., Shinagawa, M., Kim, C.J., Okamoto, Y., Minami, S., and Ogihara, K. (1999). Molecular structure of canine LINE-1 elements in canine transmissible venereal tumor. Anim. Genet. *30*, 51–53.
19. Miki, Y., Nishisho, I., Horii, A., Miyoshi, Y., Utsunomiya, J., Kinzler, K.W., Vogelstein, B., and Nakamura, Y. (1992). Disruption of the APC gene by a retrotransposal insertion of L1 sequence in a colon cancer. Cancer Res. *52*, 643–645.
20. Gilbert, N., Lutz, S., Morrish, T.A., and Moran, J.V. (2005). Multiple fates of L1 retrotransposition intermediates in cultured human cells. Mol. Cell. Biol. *25*, 7780–7795.
21. Estécio, M.R.H., Gallegos, J., Dekmezian, M., Lu, Y., Liang, S., and Issa, J.-P.J. (2012). SINE retrotransposons cause epigenetic reprogramming of adjacent gene promoters. Mol. Cancer Res. *10*, 1332–1342.
22. Han, J.S., Szak, S.T., and Boeke, J.D. (2004). Transcriptional disruption by the L1 retrotransposon and implications for mammalian transcriptomes. Nature *429*, 268–274.
23. Speek, M. (2001). Antisense promoter of human L1 retrotransposon drives transcription of adjacent cellular genes. Mol. Cell. Biol. *21*, 1973–1985.
24. Mount, S.M. (1982). A catalogue of splice junction sequences. Nucleic Acids Res. *10*, 459–472.
25. Breathnach, R., Benoist, C., O'Hare, K., Gannon, F., and Chambon, P. (1978). Ovalbumin gene: evidence for a leader sequence in mRNA and DNA sequences at the exon-intron boundaries. Proc. Natl. Acad. Sci. USA *75*, 4853–4857.
26. Vannahme, C., Gösling, S., Paulsson, M., Maurer, P., and Hartmann, U. (2003). Characterization of SMOC-2, a modular extracellular calcium-binding protein. Biochem. J. *373*, 805–814.
27. Rimbault, M., Beale, H.C., Schoenebeck, J.J., Hoopes, B.C., Allen, J.J., Kilroy-Glynn, P., Wayne, R.K., Sutter, N.B., and Ostrander, E.A. (2013). Derived variants at six genes explain nearly half of size reduction in dog breeds. Genome Res. *23*, 1985–1995.

28. Melvin, V.S., Feng, W., Hernandez-Lagunas, L., Artinger, K.B., and Williams, T. (2013). A morpholino-based screen to identify novel genes involved in craniofacial morphogenesis. Dev. Dyn. *242*, 817–831.

29. Bloch-Zupan, A., Jamet, X., Etard, C., Laugel, V., Muller, J., Geoffroy, V., Strauss, J.-P., Pelletier, V., Marion, V., Poch, O., et al. (2011). Homozygosity mapping and candidate prioritization identify mutations, missed by whole-exome sequencing, in SMOC2, causing major dental developmental defects. Am. J. Hum. Genet. *89*, 773–781.

30. Liu, P., Lu, J., Cardoso, W.V., and Vaziri, C. (2008). The SPARC-related factor SMOC-2 promotes growth factor-induced cyclin D1 expression and DNA synthesis via integrin-linked kinase. Mol. Biol. Cell *19*, 248–261.

31. Yoshida, T., Vivatbutsiri, P., Morriss-Kay, G., Saga, Y., and Iseki, S. (2008). Cell lineage in mammalian craniofacial mesenchyme. Mech. Dev. *125*, 797–808.

32. Jiang, X., Iseki, S., Maxson, R.E., Sucov, H.M., and Morriss-Kay, G.M. (2002). Tissue origins and interactions in the mammalian skull vault. Dev. Biol. *241*, 106–116.

33. Chai, Y., Jiang, X., Ito, Y., Bringas, P., Jr., Han, J., Rowitch, D.H., Soriano, P., McMahon, A.P., and Sucov, H.M. (2000). Fate of the mammalian cranial neural crest during tooth and mandibular morphogenesis. Development *127*, 1671–1679.

34. Cadieu, E., Neff, M.W., Quignon, P., Walsh, K., Chase, K., Parker, H.G., Vonholdt, B.M., Rhue, A., Boyko, A., Byers, A., et al. (2009). Coat variation in the domestic dog is governed by variants in three genes. Science *326*, 150–153.

35. Pazin, D.E., and Albrecht, K.H. (2009). Developmental expression of Smoc1 and Smoc2 suggests potential roles in fetal gonad and reproductive tract differentiation. Dev. Dyn. *238*, 2877–2890.

36. Uhlén, M., Fagerberg, L., Hallström, B.M., Lindskog, C., Oksvold, P., Mardinoglu, A., Sivertsson, Å., Kampf, C., Sjöstedt, E., Asplund, A., et al. (2015). Proteomics. Tissue-based map of the human proteome. Science *347*, 1260419.

37. Song, C., Gu, X., Feng, C., Wang, Y., Gao, Y., Hu, X., and Li, N. (2011). Evaluation of SNPs in the chicken HMGA2 gene as markers for body weight gain. Anim. Genet. *42*, 333–336.

38. Zhang, M., Pritchard, M.R., Middleton, F.A., Horton, J.A., and Damron, T.A. (2008). Microarray analysis of perichondral and reserve growth plate zones identifies differential gene expressions and signal pathways. Bone *43*, 511–520.

39. Fusco, I., Babu, D., Mellone, S., Barizzone, N., Prodam, F., Fanelli, A., Muniswamy, R., Petri, A., Bellone, S., Bona, G., and Giordano, M. (2016). Variations in the high-mobility group-A2 gene (HMGA2) are associated with idiopathic short stature. Pediatr. Res. *79*, 258–261.

40. Tetens, J., Widmann, P., Kühn, C., and Thaller, G. (2013). A genome-wide association study indicates LCORL/NCAPG as a candidate locus for withers height in German Warmblood horses. Anim. Genet. *44*, 467–471.

41. Sahana, G., Höglund, J.K., Guldbrandtsen, B., and Lund, M.S. (2015). Loci associated with adult stature also affect calf birth survival in cattle. BMC Genet. *16*, 47.

42. Liu, R., Sun, Y., Zhao, G., Wang, F., Wu, D., Zheng, M., Chen, J., Zhang, L., Hu, Y., and Wen, J. (2013). Genome-wide association study identifies Loci and candidate genes for body composition and meat quality traits in Beijing-You chickens. PLoS ONE *8*, e61172.

43. Sasaki, T., Göhring, W., Mann, K., Maurer, P., Hohenester, E., Knäuper, V., Murphy, G., and Timpl, R. (1997). Limited cleavage of extracellular matrix protein BM-40 by matrix metalloproteinases increases its affinity for collagens. J. Biol. Chem. *272*, 9237–9243.

44. Göhring, W., Sasaki, T., Heldin, C.H., and Timpl, R. (1998). Mapping of the binding of platelet-derived growth factor to distinct domains of the basement membrane proteins BM-40 and perlecan and distinction from the BM-40 collagen-binding epitope. Eur. J. Biochem. *255*, 60–66.

45. Kupprion, C., Motamed, K., and Sage, E.H. (1998). SPARC (BM-40, osteonectin) inhibits the mitogenic effect of vascular endothelial growth factor on microvascular endothelial cells. J. Biol. Chem. *273*, 29635–29640.

46. Brekken, R.A., and Sage, E.H. (2000). SPARC, a matricellular protein: at the crossroads of cell-matrix. Matrix Biol. *19*, 569–580.

47. Feng, W., Leach, S.M., Tipney, H., Phang, T., Geraci, M., Spritz, R.A., Hunter, L.E., and Williams, T. (2009). Spatial and temporal analysis of gene expression during growth and fusion of the mouse facial prominences. PLoS ONE *4*, e8066.

48. Termine, J.D., Kleinman, H.K., Whitson, S.W., Conn, K.M., McGarvey, M.L., and Martin, G.R. (1981). Osteonectin, a bone-specific protein linking mineral to collagen. Cell *26*, 99–105.

49. Maier, S., Paulsson, M., and Hartmann, U. (2008). The widely expressed extracellular matrix protein SMOC-2 promotes keratinocyte attachment and migration. Exp. Cell Res. *314*, 2477–2487.

50. Mommaerts, H., Esguerra, C.V., Hartmann, U., Luyten, F.P., and Tylzanowski, P. (2014). Smoc2 modulates embryonic myelopoiesis during zebrafish development. Dev. Dyn. *243*, 1375–1390.

51. Firth, H.V., Richards, S.M., Bevan, A.P., Clayton, S., Corpas, M., Rajan, D., Van Vooren, S., Moreau, Y., Pettett, R.M., and Carter, N.P. (2009). DECIPHER: database of chromosomal imbalance and phenotype in humans using Ensembl resources. Am. J. Hum. Genet. *84*, 524–533.

52. Alfawaz, S., Fong, F., Plagnol, V., Wong, F.S.L., Fearne, J., and Kelsell, D.P. (2013). Recessive oligodontia linked to a homozygous loss-of-function mutation in the SMOC2 gene. Arch. Oral Biol. *58*, 462–466.

53. Sulem, P., Helgason, H., Oddson, A., Stefansson, H., Gudjonsson, S.A., Zink, F., Hjartarson, E., Sigurdsson, G.T., Jonasdottir, A., Jonasdottir, A., et al. (2015). Identification of a large set of rare complete human knockouts. Nat. Genet. *47*, 448–452.

54. Maccoux, L.J., Clements, D.N., Salway, F., and Day, P.J.R. (2007). Identification of new reference genes for the normalisation of canine osteoarthritic joint tissue transcripts from microarray data. BMC Mol. Biol. *8*, 62.

55. Li, H., and Durbin, R. (2010). Fast and accurate long-read alignment with Burrows-Wheeler transform. Bioinformatics *26*, 589–595.

56. McKenna, A., Hanna, M., Banks, E., Sivachenko, A., Cibulskis, K., Kernytsky, A., Garimella, K., Altshuler, D., Gabriel, S., Daly, M., and DePristo, M.A. (2010). The Genome Analysis Toolkit: a MapReduce framework for analyzing next-generation DNA sequencing data. Genome Res. *20*, 1297–1303.

57. Ye, K., Schulz, M.H., Long, Q., Apweiler, R., and Ning, Z. (2009). Pindel: a pattern growth approach to detect break points of large deletions and medium sized insertions from paired-end short reads. Bioinformatics *25*, 2865–2871.

58. Cingolani, P., Patel, V.M., Coon, M., Nguyen, T., Land, S.J., Ruden, D.M., and Lu, X. (2012). Using *Drosophila melanogaster* as a model for genotoxic chemical mutational studies with a new program, SnpSift. Front. Genet. *3*, 35.

59. Ward, L.D., and Kellis, M. (2012). HaploReg: a resource for exploring chromatin states, conservation, and regulatory motif alterations within sets of genetically linked variants. Nucleic Acids Res. *40*, D930–D934.

60. Kircher, M., Witten, D.M., Jain, P., O'Roak, B.J., Cooper, G.M., and Shendure, J. (2014). A general framework for estimating the relative pathogenicity of human genetic variants. Nat. Genet. *46*, 310–315.

61. Purcell, S., Neale, B., Todd-Brown, K., Thomas, L., Ferreira, M.A.R., Bender, D., Maller, J., Sklar, P., de Bakker, P.I.W., Daly, M.J., and Sham, P.C. (2007). PLINK: a tool set for whole-genome association and population-based linkage analyses. Am. J. Hum. Genet. *81*, 559–575.

62. Chang, C.C., Chow, C.C., Tellier, L.C., Vattikuti, S., Purcell, S.M., and Lee, J.J. (2015). Second-generation PLINK: rising to the challenge of larger and richer datasets. Gigascience *4*, 7.

63. Falush, D., Stephens, M., and Pritchard, J.K. (2007). Inference of population structure using multilocus genotype data: dominant markers and null alleles. Mol. Ecol. Notes *7*, 574–578.

64. Zhou, X., and Stephens, M. (2012). Genome-wide efficient mixed-model analysis for association studies. Nat. Genet. *44*, 821–824.

65. Delaneau, O., Zagury, J.-F., and Marchini, J. (2013). Improved whole-chromosome phasing for disease and population genetic studies. Nat. Methods *10*, 5–6.

66. Dobin, A., Davis, C.A., Schlesinger, F., Drenkow, J., Zaleski, C., Jha, S., Batut, P., Chaisson, M., and Gingeras, T.R. (2013). STAR: ultrafast universal RNA-seq aligner. Bioinformatics *29*, 15–21.

67. Love, M.I., Huber, W., and Anders, S. (2014). Moderated estimation of fold change and dispersion for RNA-seq data with DESeq2. Genome Biol. *15*, 550.

68. Liao, Y., Smyth, G.K., and Shi, W. (2013). The Subread aligner: fast, accurate and scalable read mapping by seed-and-vote. Nucleic Acids Res. *41*, e108.

69. Robinson, J.T., Thorvaldsdóttir, H., Winckler, W., Guttman, M., Lander, E.S., Getz, G., and Mesirov, J.P. (2011). Integrative genomics viewer. Nat. Biotechnol. *29*, 24–26.

70. Schneider, C.A., Rasband, W.S., and Eliceiri, K.W. (2012). NIH Image to ImageJ: 25 years of image analysis. Nat. Methods *9*, 671–675.

71. Klingenberg, C.P. (2011). MorphoJ: an integrated software package for geometric morphometrics. Mol. Ecol. Resour. *11*, 353–357.

72. Pfaffl, M.W. (2001). A new mathematical model for relative quantification in real-time RT-PCR. Nucleic Acids Res. *29*, e45.

73. Nieto, M.A., Patel, K., and Wilkinson, D.G. (1996). In situ hybridization analysis of chick embryos in whole mount and tissue sections. Methods Cell Biol. *51*, 219–235.

74. Klingenberg, C.P., Barluenga, M., and Meyer, A. (2002). Shape analysis of symmetric structures: quantifying variation among individuals and asymmetry. Evolution *56*, 1909–1920.

75. DePristo, M.A., Banks, E., Poplin, R., Garimella, K.V., Maguire, J.R., Hartl, C., Philippakis, A.A., del Angel, G., Rivas, M.A., Hanna, M., et al. (2011). A framework for variation discovery and genotyping using next-generation DNA sequencing data. Nat. Genet. *43*, 491–498.

76. Van der Auwera, G.A., Carneiro, M.O., Hartl, C., Poplin, R., del Angel, G., Levy-Moonshine, A., Jordan, T., Shakir, K., Roazen, D., Thibault, J., et al. (2013). From FastQ data to high confidence variant calls: the Genome Analysis Toolkit best practices pipeline. Curr. Protoc. Bioinformatics *43*, 11.10.1–11.10.33.

77. Freedman, A.H., Gronau, I., Schweizer, R.M., Ortega-Del Vecchyo, D., Han, E., Silva, P.M., Galaverni, M., Fan, Z., Marx, P., Lorente-Galdos, B., et al. (2014). Genome sequencing highlights the dynamic early history of dogs. PLoS Genet. *10*, e1004016.

78. Bai, B., Zhao, W.-M., Tang, B.-X., Wang, Y.-Q., Wang, L., Zhang, Z., Yang, H.-C., Liu, Y.-H., Zhu, J.-W., Irwin, D.M., et al. (2015). DoGSD: the dog and wolf genome SNP database. Nucleic Acids Res. *43*, D777–D783.

STAR★METHODS

KEY RESOURCES TABLE

REAGENT or RESOURCE	SOURCE	IDENTIFIER
Biological Samples		
Canis familiaris	Various veterinary referral hospitals	N/A
Chemicals, Peptides, and Recombinant Proteins		
Trizol	Life Technologies	15596026
RNAlater	Life Technologies	AM7020M
Critical Commercial Assays		
CanineHD Whole-Genome Genotyping SNP BeadChip	Illumina	WG-440-1001
Truseq DNA nano kit	Illumina	FC-121-4001
TruSeq Stranded mRNA Library Prep Kit High Throughput	Illumina	RS-122-2103
Illumina TruSeq Nano DNA library prep HT	SeqLab	20000903
Deposited Data		
RNA and DNA sequencing data	This paper	ENA: PRJEB17926, http://www.ebi.ac.uk/ena
Dog reference genome (CanFam3.1, ENSEMBL release-85)	ENSEMBL	http://www.ensembl.org/index.html
Dog genotypes	This paper	http://dx.doi.org/10.5061/dryad.cq612
Dog genetic variants	Dog Biomedical Variant Database Consortium (tosso.leeb@vetsuisse.unibe.ch)	N/A
Experimental Models: Organisms/Strains		
Mouse: Smoc2tm1.1(KOMP)Vlcg	The Jackson Laboratory	https://www.jax.org
Mouse embryos	Roslin Institute Biological Research Facility	N/A
Chicken embryos	NARF	http://www.narf.ac.uk
Oligonucleotides		
gDNA targeted primer: *SMOC2* Forward: GGCAGGGG ATGGGGAAGGCT	This paper	N/A
gDNA targeted primer: *SMOC2* Reverse (ancestral): ACTGTGTGCTTTGCCCAAACTCA	This paper	N/A
gDNA targeted primer: *SMOC2* Reverse (derived): TGCCCATAAAGTTCAGGGTCCACT	This paper	N/A
gDNA targeted primer: *IGF1* Forward: CACTGATCCAG AAGAATCCAACT	[27]	N/A
gDNA targeted primer: *IGF1* Reverse: CAAAGAACCA TGTAAGCCTATTTGT	[27]	N/A
gDNA targeted primer: *STC2* Forward: ATACAATCC ACCTAGTGTCCCCAACCAT	[27]	N/A
gDNA targeted primer: *STC2* Reverse: GGCCACAGC CCCTTTAAT	[27]	N/A
gDNA targeted primer: *SMAD2* Forward: GCTTCAAG TCAGTGTGCTCC	This paper	N/A
gDNA targeted primer: *SMAD2* Reverse: CGTATTTGT TGCTGCTGGGT	This paper	N/A
gDNA targeted primer: *SMAD2* Reverse: AGAGCCCTG ACATCATGACC	This paper	N/A

(*Continued on next page*)

Continued

REAGENT or RESOURCE	SOURCE	IDENTIFIER
gDNA targeted primer: *FGF4* retrogene Forward: CACA CAGATGGACCATGAAA	This paper	N/A
gDNA targeted primer: *FGF4* retrogene Reverse (ancestral): TTTTAGATTCCGCACATGAG	This paper	N/A
gDNA targeted primer: *FGF4* retrogene Reverse (derived): CTCTTTGAACTTGCACTCCTC	This paper	N/A
gDNA targeted primer: *BMP3* Forward: GATACAGG AGATTGTGCCAAATGGGTAA	[8]	N/A
gDNA targeted primer: *BMP3* Reverse: CTCCTGGTGG AAATCGTCAGTCTATCTG	[8]	N/A
gDNA targeted primer: CFA30 QTL Forward: AGGGA TAGTCCAGCTCCAAGGCTGGTAT	This paper	N/A
gDNA targeted primer: CFA30 QTL Reverse: CTCTTTC AGGCTTCCCCAGTTGTACCTA	This paper	N/A
gDNA targeted primer: *IGF1R* Forward: AGATGACCAA CCTCAAGGATATT	[27]	N/A
gDNA targeted primer: *IGF1R* Reverse: AGTCCTGC CATCCCACAAAG	[27]	N/A
gDNA targeted primer: *GHR*(1) & *GHR*(2) Forward: GCTCTCCGTTAAATCAAGCTG	[27]	N/A
gDNA targeted primer: *GHR*(1) & *GHR*(2) Reverse: AAGGAGAGAGGTGTTGTTGGT	[27]	N/A
cDNA targeted primer: *SMOC2* Exon 2/3 Forward: TGCTTATCGAGGAAATTGCAG	This paper	N/A
cDNA targeted primer: *SMOC2* Exon 2/3 Reverse: TGGGATGAACACCTGCTGTA	This paper	N/A
cDNA targeted primer: *SMOC2* Exon 10/11 Forward: CGCGCTCTCTACCGACAT	This paper	N/A
cDNA targeted primer: *SMOC2* Exon 10/11 Reverse: GGGGTCGGGTTCTGAGAG	This paper	N/A
cDNA targeted primer: *MRPS7* Forward: AGTGCAG GGAGAAGAAGCAC	[54]	N/A
cDNA targeted primer: MRPS7 Reverse: CAGCAGCTC GTGTGACAACT	[54]	N/A
Software and Algorithms		
Read alignment: bwa v0.7.8	[55]	https://sourceforge.net/projects/bio-bwa/files/
Variant caller: GATK v3.7	[56]	http://gatkforums.broadinstitute.org/gatk
WGS utility: Picard	http://broadinstitute.github.io/picard	https://github.com/broadinstitute/picard/releases
Structural variant caller: Pindel v0.2.3	[57]	http://gmt.genome.wustl.edu/packages/pindel/
Annotation: SNPsift v4.0	[58]	http://snpeff.sourceforge.net/SnpSift.html
Effect prediction: HaploReg v4.1	[59]	N/A
Effect prediction: CADD v3.1	[60]	N/A
Utility: PLINK v1.07	[61]	http://zzz.bwh.harvard.edu/plink/
Utility: PLINK v1.90 beta	[62]	https://www.cog-genomics.org/plink2
Admixture assessments: STRUCTURE v2.3	[63]	http://web.stanford.edu/group/pritchardlab/structure_software/release_versions/v2.3.4/html/structure.html
Linear mixed model: GEMMA v0.94.1	[64]	http://www.xzlab.org/software.html
Phasing: SHAPEIT v2.r837	[65]	https://mathgen.stats.ox.ac.uk/genetics_software/shapeit/shapeit.html

(*Continued on next page*)

Continued

REAGENT or RESOURCE	SOURCE	IDENTIFIER
Graphics and data analysis: R v3.3.0	The Comprehensive R Archive Network (CRAN)	https://cran.r-project.org
RNaseq alignment: STAR v2.5.1b	[66]	https://github.com/alexdobin/STAR
RNaseq analysis: DESeq2 v1.12.14	[67]	https://bioconductor.org
RNaseq analysis: RSubread v1.22.3	[68]	https://bioconductor.org
Data visualization: Integrative Genomics Viewer v2.3.59	[69]	http://software.broadinstitute.org/software/igv/
DICOM reconstruction and landmarking: CheckPoint v2016.11.21.0711 WIN x64	Stratovan	https://www.stratovan.com
ImageJ v1.50 g	[70]	https://imagej.nih.gov/ij/
Geometric morphometrics: MorphoJ v1.06c	[71]	http://www.flywings.org.uk/morphoj_page.htm
Other		
KOD Xtreme HotStart Polymerase	Merck	71975-3
Saliva sample collection kit	Peformagene	PG-100
Lysing matrix D 2mL tube	MPBio	116913050
RNeasy Minikit	QIAGEN	C-74104
SuperScript III First- Strand Synthesis SuperMix	Life Technologies	11752050

CONTACT FOR REAGENT AND RESOURCE SHARING

Further information and requests for resources and reagents should be directed to and will be fulfilled by the Lead Contact, Jeffrey Schoenebeck (jeff.schoenebeck@roslin.ed.ac.uk).

EXPERIMENTAL MODEL AND SUBJECT DETAILS

Study Participants. In total, 374 canine patients (212 male, 162 female) were recruited from four veterinary practices across the United Kingdom and Switzerland: The Hospital for Small Animals, The University of Edinburgh, UK; Davies Veterinary Specialists, Hertfordshire, UK; Small Animal Medicine and Surgery Group, The Royal Veterinary College, Hertfordshire, UK; The Division of Clinical Radiology, The Vetsuisse Faculty University of Bern, Switzerland. Canine participants were admitted to referral practices for diagnostic imaging. Owners provided breed identity (when known) and consent for their dogs' participation in our study. Spiral or sequential computed tomography (CT) scans were acquired at one or two millimeter slice thickness. All scans were reviewed by a radiologist to ensure that pathologies or injuries did not compromise exterior skull integrity. All 374 individuals are represented in the viscerocranium and neurocranium dataset. Due to mandibular pathologies, a subset of 355 individuals were represented in the mandibular dataset. Participants were aged twenty-four months or above at the time of diagnostic imaging and represent eighty-four Kennel Club (UK) recognized breeds and eighty-three mixed-breed individuals (Table S1). Use of referral patient diagnostic imaging and biomaterial was reviewed and approved by the R(D)SVS's Veterinary Ethics Review Committee.

Mouse (C57BL/6) and chick (Isa Brown) embryos used for histology were surplus biomaterial harvested prior to this study. Mouse and chick work was conducted in accordance to animal use guidelines of the Roslin Institute under UK Home Office license and with ethical review.

METHOD DETAILS

DNA Extraction and Microarray Genotyping

Genomic DNA (gDNA) was extracted from residual diagnostic whole blood stored in EDTA at 4°C, −20°C or at −80°C; discarded soft tissue following surgery stored at −20°C; or oral saliva swabs (Performagene, DNA Genotek). DNA was extracted from whole blood using an adaption of Boodram salt-based protocol (http://www.protocol-online.org/prot/Protocols/Extraction-of-genomic-DNA-from-whole-blood-3171.html). For the gDNA extraction from soft tissue 750 μL extraction buffer (10 mM Tris pH 8.0, 10 mM EDTA pH 8.0, 1% SDS, 100 mM NaCl), 80 μL 0.5 M Dithiothreitol and 15 μL Protein K solution (20mg/mL, Ambion, Life Technologies) were added to approximately 4 mm^3 of tissue. Following overnight digestion, 270 μL saturated NaCl solution was added and centrifuged. One mL absolute ethanol was added to 500 μL supernatant to precipitate the gDNA. gDNA was spun and following centrifugation, washed with 70% ethanol. All gDNA samples were resuspended and stored in TE buffer 4°C. Oral mucosa swabs were processed in accordance with the Performagene protocol (http://www.dnagenotek.com/US/pdf/PD-PR-083.pdf). Genotypes

were produced using the 170,000 SNP Illumina CanineHD Whole-Genome Genotyping BeadChip by Edinburgh Genomics, UK. Genotype calls were mapped to CanFam3.1 coordinates (Broad, September 2011).

RNA Extraction and qPCR

Testes were selected for messenger RNA (mRNA) extraction due to the unattainability of appropriate embryonic-stage tissue or healthy adult tissues in the dog. *SMOC2* was assumed to be expressed in the testis based on evidence in other species [35, 36] (http://www.proteinatlas.org/). gDNA and mRNA were extracted from testes snap frozen and stored at -80°C in RNAlater. gDNA was extracted from tissue following the ThermoFisher Scientific protocol (http://www.thermofisher.com/uk/en/home/references/protocols/nucleic-acid-purification-and-analysis/rna-protocol/genomic-dna-preparation-from-rnalater-preserved-tissues.html). gDNA samples were genotyped for the *SMOC2* LINE-1 insertion to allow targeted extraction of RNA from testes. From our screening, we identified nine subjects: 3 ancestral (1 Italian greyhound, 1 whippet, 1 Yorkshire terrier), 3 heterozygous (1 Papillon, 2 Cavalier King Charles spaniels), and 3 homozygous derived (1 bulldog, 1 French bulldog, 1 pug). For RNA extractions, 1 mL chilled Trizol was added to 100 mg of testes in a matrix D lysis tube and homogenized using a FastPrep for two 20 s intervals at 4 m/s. Samples were incubated at room temperature for 5 min following homogenization. Next, 200 μL 1-bromo-3-chloropropane (BCP) was added to each sample, shaken vigorously for 15 s and incubated at room temperature for 3 min. Samples were centrifuged for at 12,000 G for 15 min at 4°C and the upper aqueous phase was subsequently transferred to a fresh tube. RNA was cleaned using the QIAGEN RNeasy Minikit following and including optional steps provided in the RNeasy Mini Kit Part 1 protocol. A DNase step was not used.

Complementary DNA (cDNA) was produced from 1 μg total RNA using the SuperScript III First-Strand Synthesis SuperMix (Invitrogen) following the product protocol with oligo(dT) primers. Primers for target genes were designed to be intron-spanning using the online Roche design center. Primers for reference housekeeping genes were acquired from previously published work [54]. Relative expression profiles for *SMOC2* were determined using the Roche Life Sciences probe-based real-time qPCR assay with a LightCycler 480 system (Roche). All RNA profiles were analyzed in triplicate for both technical and biological replicates. Expression of target genes were normalized with mitochondrial ribosomal protein S7 (MRPS7). Relative quantification levels were corrected for primer efficiency [72].

Sequencing Library Preparations

The integrity of genomics DNA and total RNA samples were verified by Agilent Tapestation. All RNA samples scored RIN values greater than 8.0. DNA and RNA Library preparation and sequencing services were provided by Edinburgh Genomics (UK). Briefly, DNA libraries were prepared using either SeqLab TruSeq Nano DNA library prep HT or Illumina Truseq DNA nano DNA library kits. Paired-end libraries sequences on the Illumina HiSeq 2500 had an average insert size of 550 bp and read length of 125 bp. Paired-end DNA libraries sequenced on the HiSeq X platform had an average insert size of 450 bp and 150 bp read length.

For RNA, TruSeq stranded libraries were prepared from nine preparations of total RNA according to manufacturer's protocol. Barcoded libraries were sequenced on three lanes of an Illumina HiSeq 4000, producing 150 bp paired-end reads (96 million + 96 million reads per library).

Histology

Whole-mount *Smoc2 in situ* hybridization was performed per Nieto et al. (1996) [73].

QUANTIFICATION AND STATISTICS

Morphometrics

3D reconstructions of anonymised canine skull CT scans were generated in Stratovan Checkpoint software (v2014.11.28.0324) and anatomical substructures (cranium and mandible) of resulting isosurfaces were manually landmarked by a single analyst (Figure S1). Breed designations were hidden from the analyst and CTs were analyzed in a random order. Fifty-six cranial and thirty mandibular landmarks were selected to capture morphological variation. Raw 3D coordinates of cranial and mandibular subsets were reformatted using custom R (v3.2.5) scripts and analyzed using MorphoJ (v1.06c) [71]. The cranial landmark subset was further divided into neurocranium (n = 18) and viscerocranium (n = 25) landmarks (Figure S1). A generalized Procrustes fit was used to scale, transpose, and rotate landmarks [74]. A by-product of the Procrustes fit is the centroid size (the amount of scaling used in the fit). The neurocranium centroid size was used as a proxy of body size (see below). In order to remove allometric effects, a regression consisting of 10,000 permutations using the neurocranium's centroid size (independent variable) was run on the viscerocranium and mandible symmetric coordinates. A covariance matrix was calculated from the regression residuals. Decomposition of the distance matrix by principal component analysis (PCA) produced components; each principal component (PC) explains successively smaller tranches of morphological variation. Viscerocranium PC1 (without allometry), mandible PC1 (without allometry) and neurocranium centroid size were subsequently used as phenotypic outcomes for GWAS.

Lateral and dorsoventral radiographs of four C57BL/6JN background controls and eight *Smoc2*$^{-/-}$ mice (*Smoc2*$^{tm1.1(KOMP)Vlcg}$ allele produced by The Jackson Laboratory, USA) aged thirteen weeks were landmarked in ImageJ (v1.50 g) [70] using the PointPicker plugin (male = 5, female = 7). The raw 2D coordinates for nine lateral and fifteen dorsoventral landmarks were exported from ImageJ and analyzed in MorphoJ. Lateral and dorsoventral landmarks were analyzed as using the same approach. A generalized Procrustes fit was used to create a best fit for landmarks. A covariance matrix was calculated using the Procrustes

distance matrix of the whole head prior to PCA. A two-tailed Student's t test assessed PC1 distribution for sex and *Smoc2* background. Principal component plots were generated using a custom R script. Additional phenotypic detail regarding these mice are available form the International Mouse Phenotyping Consortium (http://www.mousephenotype.org).

Genotype Analyses

PLINK (v1.07) [61] was used to remove SNPs with a minor-allele frequency < 0.05 and individuals with > 0.1 missing markers. Genotypes were prephased using SHAPEIT (v2.r837) [65] using default parameters that includes 500 states. Imputations were done with Minimac2 (2014.9.15) using 40 rounds and 1,000 states. Post-processing by fcGene (v1.0.7) removed genotypes with $R^2 < 0.3$ and minor allele frequency < 0.05. In total, 139,260 SNPs remained for analysis.

Population structure was assessed using STRUCTURE (v2.3) [63]. GEMMA (v0.94.1) [64], which incorporates a kinship matrix in its implementation of univariate linear mixed models, was used to perform genome-wide association tests. Sex and up to ten principal components (generated from SNP genotype data in PLINK v1.9 [62]) were used as covariates – ten covariates for neurocranium and five for viscerocranium and mandible. The number of PCs included was determined by evaluation of Q-Qplots. p values generated in the association tests were used for Q-Qplots; using the aforementioned parameters returned λ values (genomic inflation factors) within the range 0.954 – 1.000 (Figure 2 and Figure S4). Index SNPs as well as markers in linkage disequilibrium ($r^2 > 0.2$) to them were pruned from the dataset using PLINK and GEMMA association analyses were re-run. Observed p values plotted concordantly with expected values, indicating minimal population-based inflation. A Bonferroni correction was used to determine a significance threshold for association tests ($-\log_{10}[0.05/139,260] = 6.44$). Manhattan plots and Q-Qplots were generated using custom scripts in R.

Fine Mapping

Haplotype association testing was done using ten SNP sliding windows across ~1 megabase (Mb) flanking regions of significant SNPs in canine chromosome (CFA) 1. SHAPEIT was used to phase genotypes. Haplotypes for the region of interest were ordered by individual viscerocranium PC1 score and colored by genotypes that matched the consensus sequence. The borders of the critical interval were defined by a minimum of three meiotic recombination events across the brachycephalic individuals with a viscerocranium PC1 < −0.2.

Variant Filtering

Eight brachycephalic dogs were resequenced on an Illumina HiSeq 2000 (Edinburgh Genomics, UK) to approximately 14-33X depth. Another thirty dogs were resequenced using the Illumina HiSeq X platform to > 40X depth. Resulting paired-end reads were aligned to the reference genome (CanFam3.1, Broad September 2011) using bwa (v0.7.8) [55]. SNPs and small INDEL variants within the critical interval (CFA1:55850299-56037676) were called using GATK (v3.7) [56, 75, 76]. We compared our variant calls to those of three-hundred and four dogs and wild canids made available to the DBVCD consortium members and an additional five canids (1 Basenji, 4 wolves) from the DoGSD database [77, 78]. Variants were hard filtered using SnpSift (v4.0) [58]. Because their deep coverage and large insert sizes (> 450bp), we used our thirty-eight re-sequenced dogs to call structural variants; variants were called using Pindel (v0.2.3) [57]. Filtering criteria for both SNPs/INDELS and large structural variants were determined by the presence of the twelve SNP haplotype across selected brachycephalic and dolichocephalic individuals whose skull phenotypes were confirmed (Table S4). Our filtering criteria were based on five logical assumptions. First, genomes from brachycephalic dogs with the twelve SNP haplotype were assumed to carry, or to be fixed for, the causal variant(s) within the CFA1 critical interval. Second, haplotype sharing at the CFA1 locus suggests identity-by-descent; therefore brachycephalic dogs with the twelve SNP haplotype inherited the same causal variant(s) from a common ancestor. Third, as the dog assembly is based on the genome of a boxer (a brachycephalic dog that was fixed for the twelve SNP haplotype), the causal variant(s) could be present in the reference assembly as reference allele(s). Fourth, we expected that the causal variant(s) are derived and therefore absent from wild canid populations such as dogs' ancestor, the gray wolf. Lastly, dolichocephalic dogs without the associated twelve SNP haplotype cannot carry the causal variant(s).

qRT-PCR

All RNA profiles were analyzed in triplicate for both technical and biological replicates. Expression of target genes were normalized with mitochondrial ribosomal protein S7 (MRPS7). Relative quantification levels were corrected for primer efficiency [72].

RNA-Seq

FASTQ files were aligned using STAR to the dog reference genome (CanFam3.1, ENSEMBL release-85). Annotated junctions were downloaded from ENSEMBL (ftp.ensembl.org/pub/release-85/gtf/canis_familiaris/Canis_familiaris.CanFam3.1.85.gtf.gz). Alignment was performed in two passes as instructed by the user manual. Using Picard tools (http://broadinstitute.github.io/picard), read groups were added, bam files were merged by sample, and reads were marked for duplicates. Using featureCounts, an analysis tool of the RSubread package (RSubread v1.22.3 installed on R v3.3.0), we quantified mapped reads to genes. Differential expression analysis was conducted at the gene level using the R package DESeq2 (v1.12.4) by comparing homozygous *SMOC2* LINE-1 carriers

compared to non-carriers (three each). Detection of allelic imbalance was made possible by two of the three heterozygous dogs described above (1 Cavalier King Charles spaniel, 1 Papillon), as these dogs were also heterozygous for the C/T SNP in exon 8 (chr1:55939143) of *SMOC2*.

Generation Scotland: Scottish Family Health Study

We used whole exome sequences from the Generation Scotland: Scottish Family Health Study (GS:SFHS). Study participants had been originally recruited for population-based studies of complex traits. Details regarding the design and sequencing of human participants is described elsewhere. We extracted all sequence variants in *SMOC2* which passed GATK recalibration [56]. Putative regulatory elements and functional roles of the extracted variants were assessed by the ENCODE-based prediction tool HaploReg (v4.1) [59]. To assess the predicted consequences of the variants, we examined their C-scores, which indicate the 'deleteriousness' of a given mutation using combined annotation dependent depletion (CADD, v3.1) [60].

DATA AND SOFTWARE AVAILABILITY

DNA-seq and RNA-seq data are publicly available at the European Nucleotide Archive under primary accession number ENA: PRJEB17926. Genotypes are available at Dryad Digital Repository (http://datadryad.org). The Dryad Digital Repository DOI for the genotype data reported in this paper is Dryad: 10.5061/dryad.cq612.

ADDITIONAL RESOURCES

DECIPHER

This study makes use of data generated by the DECIPHER community. A full list of centers who contributed to the generation of the data is available from https://decipher.sanger.ac.uk and via email from decipher@sanger.ac.uk. The DECIPHER database was searched for variants in human *SMOC2* with reported craniofacial phenotypes.

Current Biology, Volume *27*

Supplemental Information

Canine Brachycephaly Is Associated with a Retrotransposon-Mediated Missplicing of *SMOC2*

Thomas W. Marchant, Edward J. Johnson, Lynn McTeir, Craig I. Johnson, Adam Gow, Tiziana Liuti, Dana Kuehn, Karen Svenson, Mairead L. Bermingham, Michaela Drögemüller, Marc Nussbaumer, Megan G. Davey, David J. Argyle, Roger M. Powell, Sérgio Guilherme, Johann Lang, Gert Ter Haar, Tosso Leeb, Tobias Schwarz, Richard J. Mellanby, Dylan N. Clements, and Jeffrey J. Schoenebeck

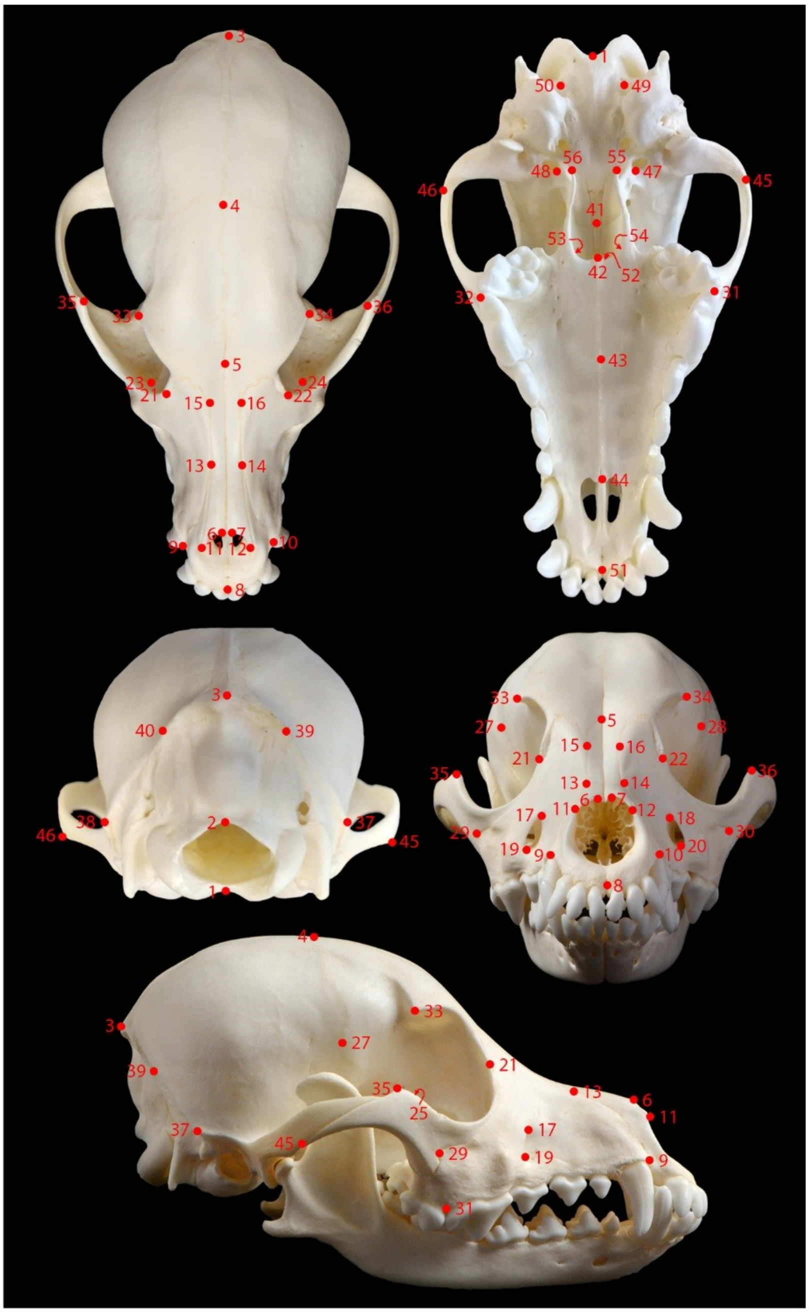

Skull Landmarks

1. Basion (Brainstem basis) *
2. Opisthion *
3. Inion *
4. Bregma *
5. Nasion * †

6/7. Internasal Suture (R+L) †

8. Prosthion †

9/10. Incisive-Maxilla Junction with Alveolus (R+L) †
11/12. Nasal Bone Process (R+L) †
13/14. Incisive-Maxilla-Nasal Junction (R+L) †
15/16. Frontal-Maxilla-Nasal Junction (R+L) †
17/18. Dorsal Aspect of the Infraorbital Foramen (R+L) †
19/20. Ventral Aspect of the Infraorbital Foramen (R+L) †
21/22. Frontal-Lacrimal-Maxilla Junction (R+L) †
23/24. Dorsal Aspect of the Maxillary Foramen (R+L)
25/26. Dorsal Aspect of the Ethmoid Foramina (R+L)
27/28. Pterion (R+L) *
29/30. Zygomatic Process of the Maxilla (R+L) †
31/32. Molar-Premolar Interalveolar Septa (R+L) †
33/34. Zygomatic Process of the Frontal Bone (R+L) *
35/36. Frontal Process of the Zygomatic Bone (R+L)
37/38. Nuchal Crest Flare (R+L) *
39/40. Asterion (R+L) *

41. Anterior Hirnstammbasis *
42. Posterior Nasal Spine
43. Maxilla-Palatine Junction at Midline †
44. Incisive-Maxilla Junction at Midline †

45/46. Ventral Temporal-Zygomatic Junction (R+L)
47/48. Rostral Aspect of the Foramen Ovale (R+L) *
49/50. Rostral Aspect of the Hypoglossal Canal (R+L) *

51. Intraoral Midline of Incisive Bone at Alveolus †
52. Midline of Vomer Dorsal to Nasal spine

53/54. Midline of Choana Dorsal to Posterior Nasal Spine (L+R)
55/56. Pterygoid Hamulus (L+R)

Mandible Landmarks

1/2. Dorsal Aspect of the Angular Process (L+R)
3/4. Lateral Aspect of the Condylar Process (L+R)
5/6. Medial Aspect of the Condylar Process (L+R)
7/8. Dorsal Aspect of the Mandibular Notch (L+R)
9/10. Dorso-Caudal Aspect of the Coronoid Process (L+R)
11/12. 3rd Molar Caudal-midline at Alveolus (L+R)
13/14. Ventral Aspect of the Masseteric Fossa (L+R)
15/16. 1st Molar Lateral-midline at Alveolus (L+R)
17/18. Canine Tooth Caudal-midline at Alveolus (L+R)
19/20. Caudal Aspect of Caudal Mental Foramina (L+R)
21/22. Caudal Aspect of Middle Mental Foramina (L+R)
23/24. Caudal Aspect of Rostral Mental Foramina (L+R)
25/26. Ventral Aspect of Inter-Mandibular Joint (L+R)
27/28. Rostral Aspect of the Mandibular Foramen (L+R)
29/30. Mid-Coronoid Process (L+R)

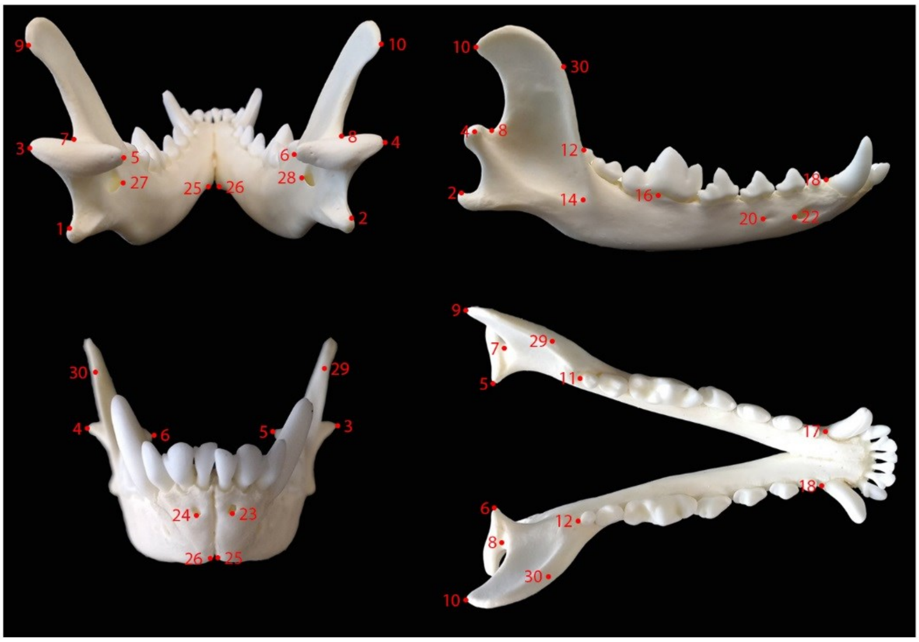

Figure S1. Landmarks of the skull and Mandible. Related to Figure 1, 2 and S3. Images illustrate the position of landmarks placed on skull isosurfaces. Skull images clockwise from top-left: Dorsal, ventral, rostral, lateral (right side) and caudal views. Mandible images clockwise from top-left: Caudal, lateral (right side), dorsal and rostral views. The canine anatomic denominations correspond to a human skull as follows: Rostral = Anterior, Caudal = Posterior, Ventral = Inferior, Dorsal = Superior. Skull landmarks are subdivided into neurocranium (*) and viscerocranium (†) datasets.

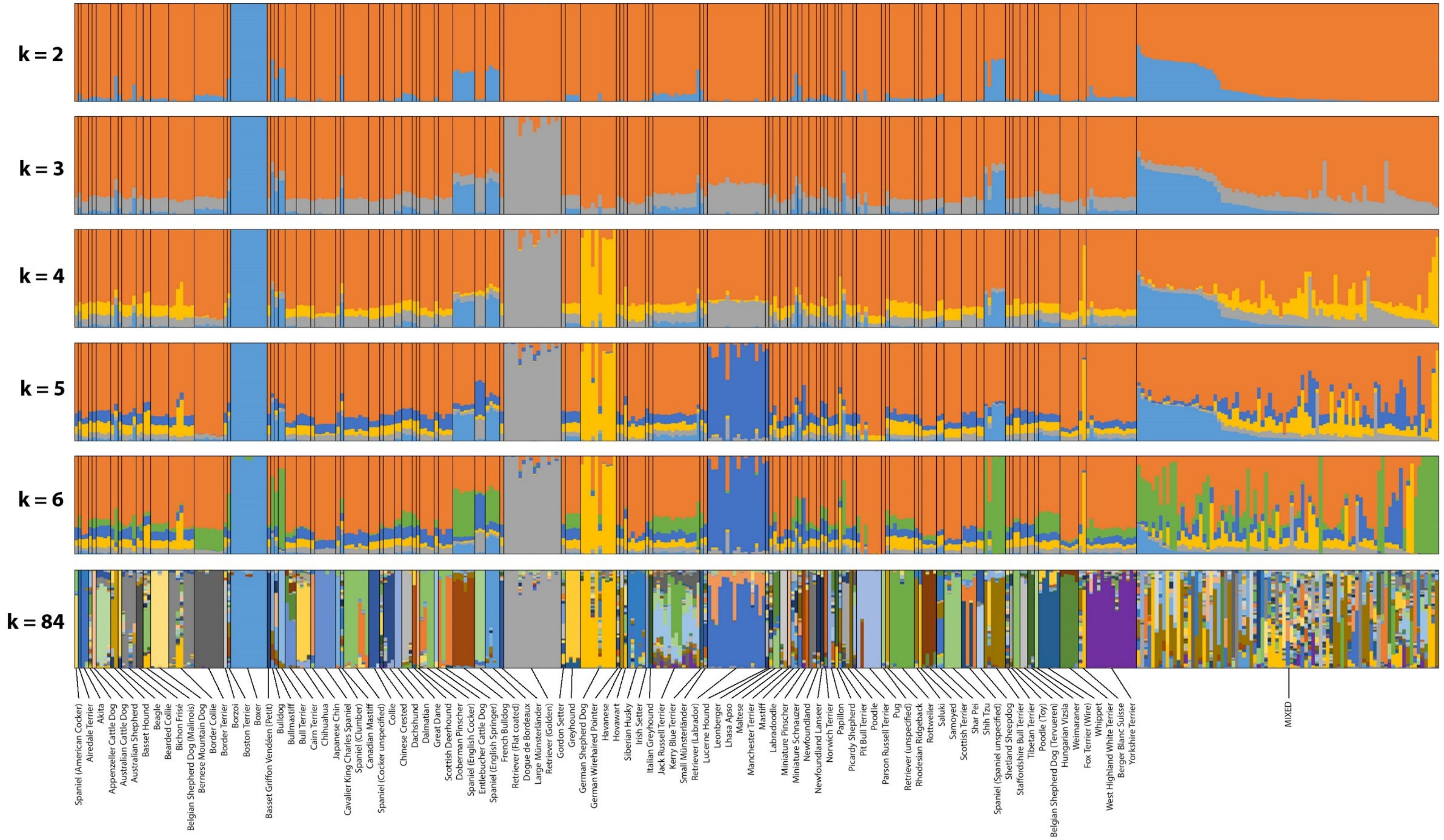

Figure S2. The genomic structure confirms the correct assignment of breed identities and infers the breed composition of mixed-breed dogs. Related to Figure 1 and 3. Population structure of all 374 individuals in this study. Each vertical bar represents an individual dog. Dogs are ordered by breed (vertical black lines). Vertical bars are split into different colours depending on the proportional membership of an individual dog to each cluster.

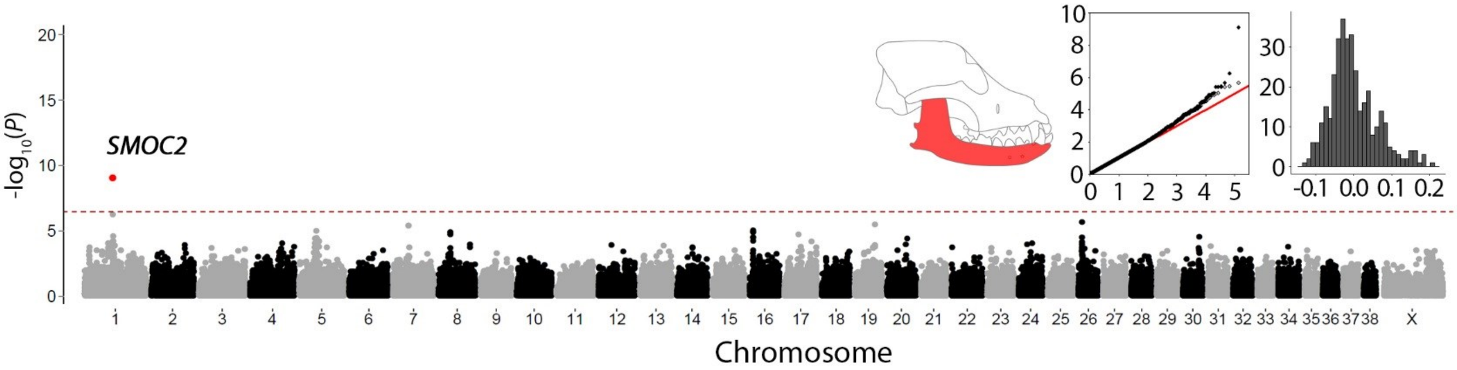

Figure S3. Variation in mandible shape is associated with a QTL on CFA1. Related to Figure 2 and S1. Manhattan plot for mandible PC1 was generated using a linear mixed model in GEMMA software. The mandible dataset was regressed using the neurocranium centroid to remove the effects of allometry. Sex and five principal components computed from SNP data were used as covariates. SNPs remaining significant following a Bonferroni correction (3.6×10^{-7}, dashed line) are coloured red. Significant SNPs are summarised in Table S3. Inserts: skull schematics indicate the origins of landmarks used for dataset. Expected (x-axis) and observed (y-axis) $-\log_{10}(P)$ values are plotted for all SNPs (black circles) and pruned SNPs (grey circles). Histogram depicts the frequency (y-axis) of mandible PC1.

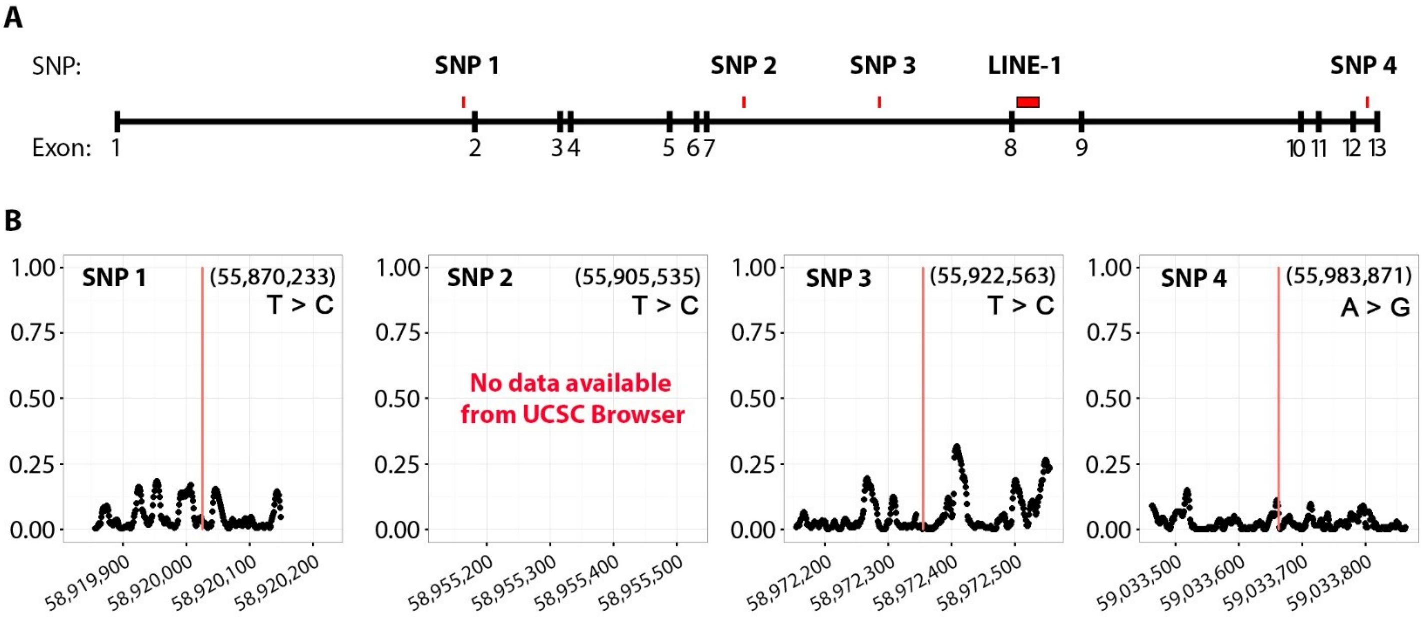

Figure S4. Remaining intronic candidate SNPs are poorly conserved across species. Related to Table 2. (A) All four remaining SNPs in the viscerocranium PC1 critical interval following filtering are located within the *SMOC2* gene. (B) Coordinates of each SNP (listed as CanFam3.1 in brackets) are converted to CanFam2.0 and used to plot PhastCon scores (y-axis) for 200 bp in both directions from the SNP (x-axis). PhastCon scores were downloaded from the UCSC Table Browser. No PhastCon scores were available from the UCSC Table Browser for SNP2. From the available data, no SNPs reside in highly conserved positions across dog, human, mice and rat genomes. Alleles are written as Ancestral > Derived.

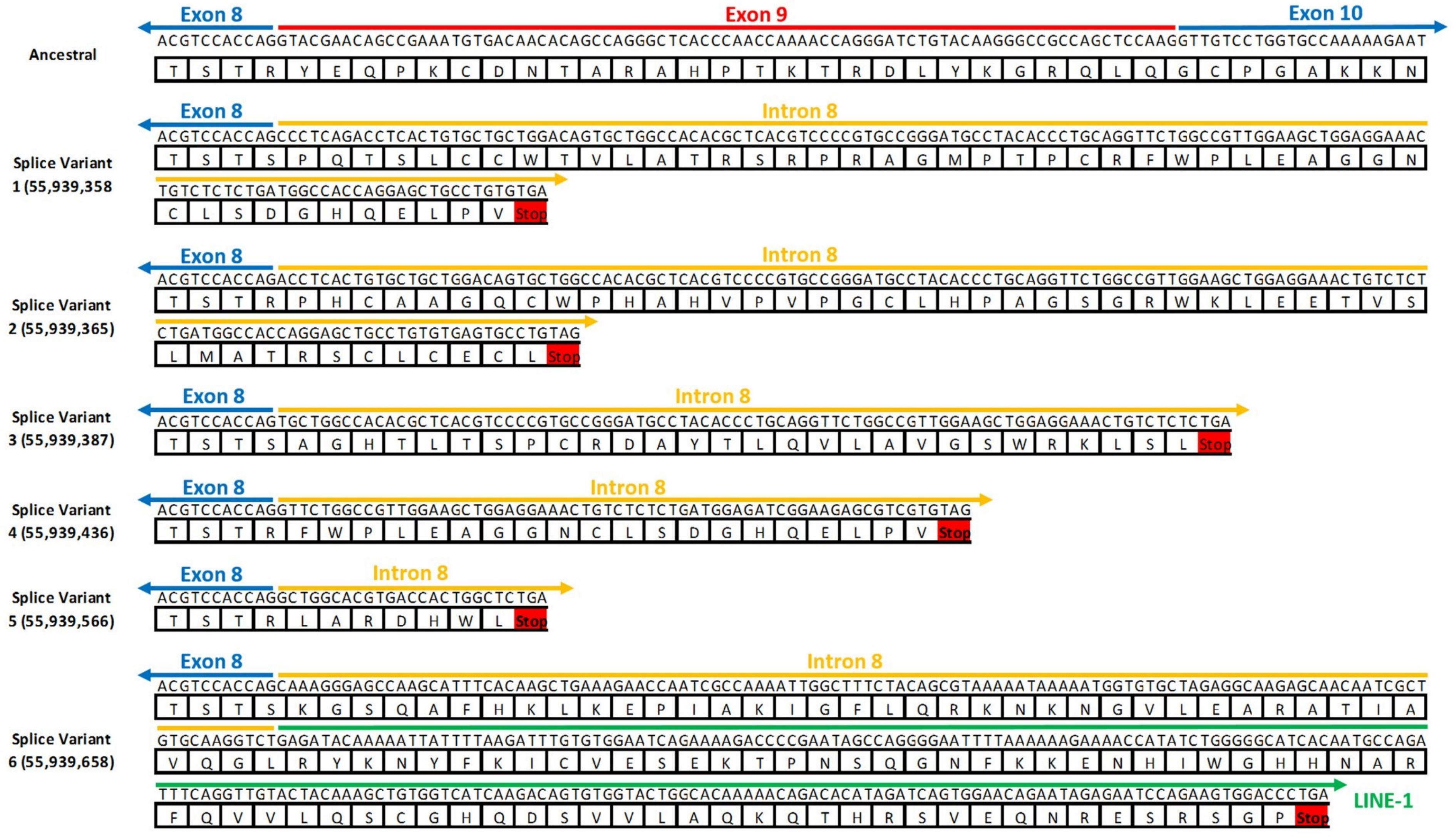

Figure S5. ***SMOC2* Isoform Sequences.** Related to Figure 4. mRNA and amino acid sequences for the canonical (ancestral) *SMOC2* transcript and the six isoforms identified in dogs that are carriers of the LINE-1 element. Base pair positions in brackets indicate the position of the first nucleotide of intron 8 to be included in the mature mRNA transcript. All isoforms are predicted to code for premature stop codons.

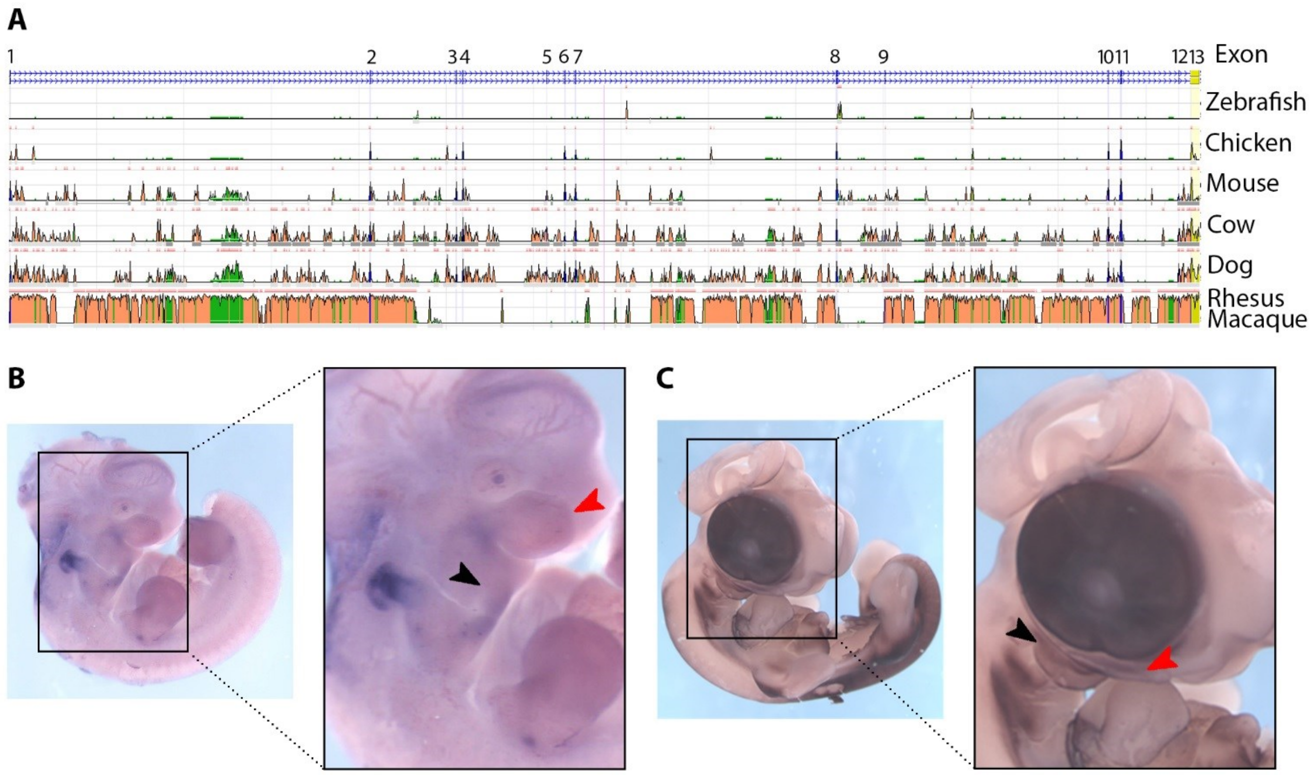

Figure S6. Evolutionary Conservation of *SMOC2*. Related to Figure 3. The Evolutiponary Conserved Regions of human *SMOC2* spanning chr6:168,851,429-169,078,272 (hg19 construct) are poorly conserved in the zebrafish, chicken and mouse compared to the dog, cow and rhesus monkey (A). Despite this, *Smoc2* mRNA is expressed in the first pharyngeal arch of the mouse and chicken. Whole mount in situ hybridisations for *Smoc2* in mouse (**B**) and chicken (**C**) embryos. *Smoc2* expression is observed in the mandibular process (black arrow) and maxillary process (red arrow) of the first pharyngeal arch.

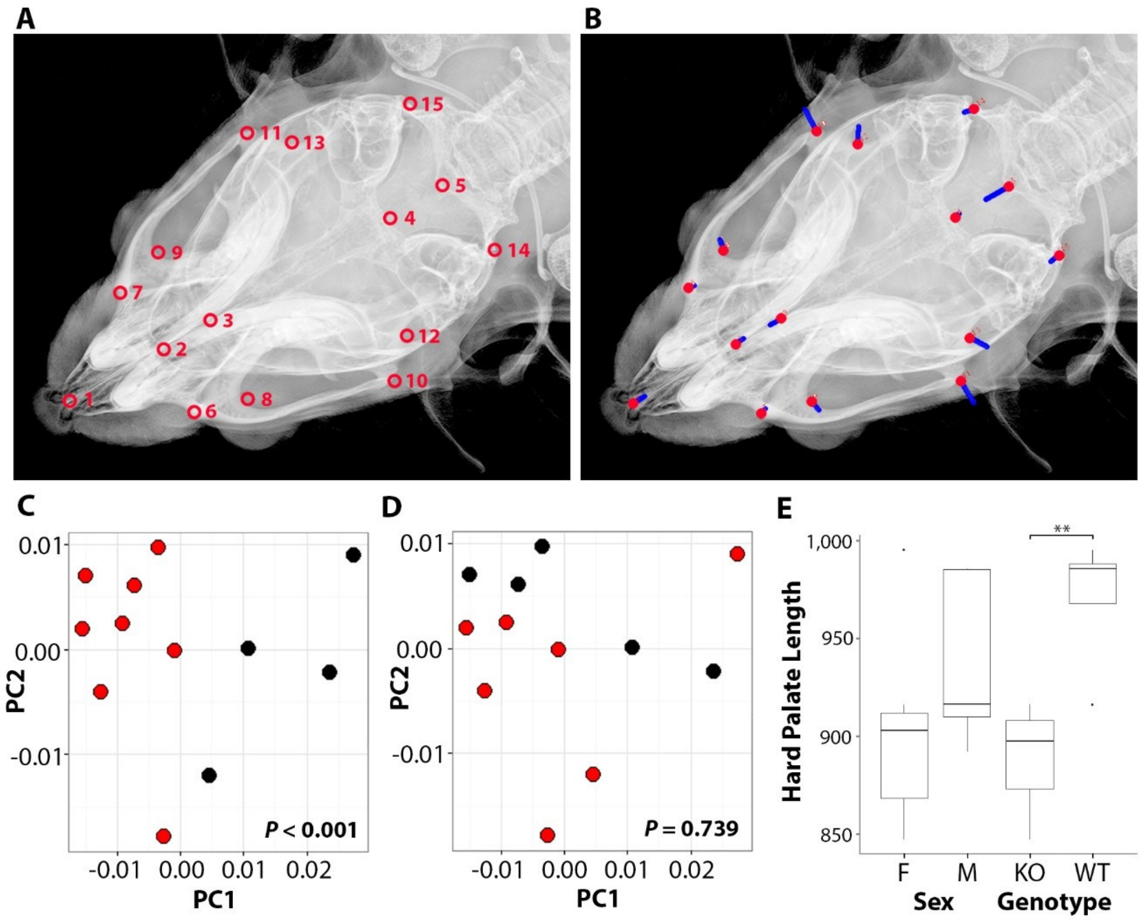

Figure S7. *Smoc2* null mice have dysmorphic skulls. Related to Figure 4. (A) Dorsoventral radiographs of mice aged 13 weeks indicating the position of fifteen landmarks across the skull. (B) Lollipop diagram denotes the mean position of landmarks (red circles) and the extent and direction of landmark variation for whole head PC1 (blue bars). The whole skull PC1 values for *Smoc2* null (red) and control mice (black) segregate significantly by (C) genotype but not (D) sex.

Abbreviation	Breed Name	Kennel Club (UK) Recognised	Viscerocranium Dataset	Mandible Dataset
ACKR	Spaniel (American Cocker)	Yes	1	1
AIRT	Airedale Terrier	Yes	1	1
AKIT	Akita	Yes	2	2
AUCD	Australian Cattle Dog	Yes	1	1
AUSS	Australian Shepherd	Yes	4	4
BASS	Basset Hound	Yes	2	2
BEAG	Beagle	Yes	1	1
BERD	Bearded Collie	Yes	4	4
BICH	Bichon Frisé	Yes	2	1
BMAL	Belgian Shepherd Dog (Malinois)	Yes	2	2
BMD-	Bernese Mountain Dog	Yes	5	5
BORD	Border Collie	Yes	7	6
BORT	Border Terrier	Yes	8	7
BORZ	Borzoi	Yes	1	1
BOST	Boston Terrier	Yes	1	1
BOX-	Boxer	Yes	10	10
BRGR	Basset Griffon Vendéen (Petit)	Yes	1	1
BULD	Bulldog	Yes	1	1
BULM	Bullmastiff	Yes	1	1
BULT	Bull Terrier	Yes	2	2
CAIR	Cairn Terrier	Yes	3	2
CHIH	Chihuahua	Yes	4	3
CHIN	Japanese Chin	Yes	1	1
CKCS	Cavalier King Charles Spaniel	Yes	6	6
CLSP	Spaniel (Clumber)	Yes	1	1
COLL	Collie	Yes	3	3
CRES	Chinese Crested	Yes	1	0
DACH	Dachshund	Yes	3	2
DALM	Dalmatian	Yes	2	2
DANE	Great Dane	Yes	3	3
DEER	Scottish Deerhound	Yes	1	1
DOBP	Doberman Pinscher	Yes	1	1
ECKR	Spaniel (English Cocker)	Yes	3	3
ENTL	Entlebucher Cattle Dog	Yes	1	1
ESSP	Spaniel (English Springer)	Yes	4	3
FBUL	French Bulldog	Yes	6	6
FCR-	Retriever (Flat coated)	Yes	3	3
FMAS	Dogue de Bordeaux	Yes	4	4
GMLD	Large Münsterländer	Yes	1	1
GOLD	Retriever (Golden)	Yes	16	15
GORD	Gordon Setter	Yes	1	1
GREY	Greyhound	Yes	4	3
GSD-	German Shepherd Dog	Yes	10	10
GWHP	German Wirehaired Pointer	Yes	1	1
HAVA	Havanese	Yes	1	1
HOVA	Hovawart	Yes	1	1
HUSK	Siberian Husky	Yes	5	5
IRSE	Irish Setter	Yes	1	1
ITGY	Italian Greyhound	Yes	1	1
JACK	Jack Russell Terrier	Yes	13	13
KERY	Kerry Blue Terrier	Yes	1	1
KMLD	Small Münsterländer	Yes	1	1
LAB-	Retriever (Labrador)	Yes	16	14
LEON	Leonberger	Yes	1	1
LHSA	Lhasa Apso	Yes	2	2
MALT	Maltese	Yes	2	2
MANT	Manchester Terrier	Yes	1	1
MAST	Mastiff	Yes	2	2

MPIN	Miniature Pinscher	Yes	2	2
MSNZ	Miniature Schnauzer	Yes	2	2
NEWF	Newfoundland	Yes	1	1
NOWT	Norwich Terrier	Yes	1	1
PAPI	Papillon	Yes	2	1
PICA	Picardy Shepherd	Yes	1	1
PRUS	Parson Russell Terrier	Yes	1	1
PUG-	Pug	Yes	7	7
RHOD	Rhodesian Ridgeback	Yes	1	1
ROTT	Rottweiler	Yes	7	7
SALU	Saluki	Yes	1	1
SAMO	Samoyed	Yes	1	1
SCOT	Scottish Terrier	Yes	4	3
SHAR	Shar Pei	Yes	2	2
SHIH	Shih Tzu	Yes	5	5
SSHP	Shetland Sheepdog	Yes	2	2
STAF	Staffordshire Bull Terrier	Yes	6	6
TIBT	Tibetan Terrier	Yes	1	1
TPOO	Poodle (Toy)	Yes	1	1
TURV	Belgian Shepherd Dog (Tervueren)	Yes	2	2
VIZS	Hungarian Vizsla	Yes	2	2
WEIM	Weimaraner	Yes	2	2
WFOX	Fox Terrier (Wire)	Yes	1	1
WHIP	Whippet	Yes	6	6
WHWT	West Highland White Terrier	Yes	5	3
YORK	Yorkshire Terrier	Yes	14	12
APPH	Appenzeller Cattle Dog	No	1	0
CKSP	Spaniel (Cocker unspecified)	No	7	7
CMAS	Canadian Mastiff	No	1	1
LBDO	Labradoodle	No	1	1
LCHO	Lucerne Hound	No	1	1
MIXED	Mixed Breed	No	83	83
NEWL	Newfoundland Lanseer	No	1	1
PITB	Pit Bull Terrier	No	1	1
POOD	Poodle	No	3	3
RTVR	Retriever (unspecified)	No	1	1
SPSP	Spaniel (Spaniel unspecified)	No	4	4
WSSD	Berger Blanc Suisse	No	2	2
		**Total:**	374	355

Table S1. Breed Demographics and Abbreviations. Related to Figure 1. Summary of the breed designation of all 374 individuals in the viscerocranium dataset and 355 individuals in the mandible dataset. Eighty-four breeds are recognised by the Kennel Club (UK).

Dataset	CFA	SNP	Position	Candidate Gene	Allele	Beta	*P*-value	Reference
Viscerocranium	1	BICF2P250912	55,983,871	***SMOC2***	G > A	-7.04E-02	1.91E-20	
		BICF2P1251361	55,862,036	***SMOC2***	C > T	-3.37E-02	1.39E-09	
		BICF2P1290166	56,132,332	*SMOC2*	A > G	-3.31E-02	1.64E-07	
Mandible	1	BICF2P250912	55,983,871	***SMOC2***	G > A	2.37E-02	8.43E-10	
Neurocranium	3	TIGRP2P56799_rs8666557	91,103,945	*LCORL/NCAPG*	G > T	1.02E+01	3.64E-09	[S1]
		BICF2G630361702	91,114,590	*LCORL/NCAPG*	G > A	1.03E+01	8.31E-09	
	7	BICF2S23352941	43,719,549	***SMAD2***	A > G	-1.11E+01	5.71E-13	[S1, S2, S3]
		BICF2S23762784	43,702,273	***SMAD2***	G > A	-1.03E+01	8.17E-12	
		BICF2P884876	43,569,230	*SMAD2*	G > C	-7.22E+00	1.65E-07	
		BICF2S23719803	43,865,905	*SMAD2*	T > C	7.64E+00	2.60E-07	
	10	G580f46S240	8,183,593	***HMGA2***	C > T	-1.41E+01	3.06E-15	[S1, S2, S3]
		BICF2P776298	8,454,499	*HMGA2*	A > G	-1.01E+01	4.36E-13	
		BICF2S23211087	7,993,147	*HMGA2*	T > C	-1.00E+01	2.77E-11	
		BICF2P283272	7,996,770	*HMGA2*	G > A	-1.00E+01	2.77E-11	
		BICF2P462764	8,772,534	*HMGA2*	T > C	-9.73E+00	3.78E-11	
		BICF2P1118840	7,957,984	*HMGA2*	T > C	-9.97E+00	3.86E-11	
		BICF2P263265	8,162,459	***HMGA2***	T > C	-9.24E+00	1.32E-09	
		BICF2P718692	8,070,103	*HMGA2*	C > T	9.40E+00	4.76E-09	
		BICF2S24410053	7,892,540	*HMGA2*	C > A	-8.21E+00	8.81E-08	
		BICF2P712340	7,884,978	*HMGA2*	T > C	8.60E+00	9.34E-08	
		BICF2S23633421	8,085,469	*HMGA2*	T > C	8.43E+00	1.01E-07	
		BICF2P797505	8,418,771	*HMGA2*	G > C	8.02E+00	1.50E-07	
		BICF2S23128717	8,779,119	*HMGA2*	G > A	7.04E+00	1.89E-07	
	15	BICF2P355320	41,257,020	***IGF1***	C > T	-1.63E+01	1.73E-19	[S1, S2, S3]
		BICF2P971192	41,221,438	***IGF1***	A > G	1.18E+01	4.38E-18	
		BICF2P893982	41,216,597	***IGF1***	A > G	1.16E+01	1.63E-17	
		BICF2P885652	41,232,547	***IGF1***	C > T	-1.04E+01	1.18E-15	
		BICF2P1107998	41,247,955	***IGF1***	C > T	-1.02E+01	1.30E-15	
		BICF2P355319	41,256,949	***IGF1***	A > G	1.01E+01	2.66E-15	
		BICF2P67088	41,206,514	***IGF1***	C > A	9.95E+00	2.23E-12	
		BICF2P117496	41,250,986	***IGF1***	C > T	9.45E+00	6.12E-11	
		BICF2P1100250	41,263,373	*IGF1*	A > C	9.12E+00	6.12E-11	
		BICF2P1292476	41,177,094	***IGF1***	T > C	7.27E+00	3.27E-08	
		BICF2P577974	41,314,290	*IGF1*	G > A	-6.88E+00	3.15E-07	
	18	BICF2S23615757	20,272,961	*FGF4* retrogene	A > C	-1.14E+01	3.31E-08	[S1, S4]
		BICF2S2323033	20,310,833	*FGF4* retrogene	T > G	-8.90E+00	2.72E-07	

Table S2. Significant SNPs. Related to Figure 2, S3 and Table 1. All significant SNPs detected in viscerocranium PC1, mandible PC1 and neurocranium centroid size datasets (Figure 2, Supplementary Figure 4). Candidate genes for each locus are listed. Gene names are highlighted in bold for intragenic SNPs.

Table S3. CFA1 Critical Interval Haplotype Summary.

See Supp_Table_3_Haplotype_Summary_2017.xlsx which is available for download.

Individual	Criteria	SnpSift (SNPs/INDELS)	Pindel (Structural variants)
American Bulldog	Homozygous Reference (Derived)	✓	✓
Brussels Griffon	Homozygous Reference (Derived)	✓	✓
French Bulldog	Homozygous Reference (Derived)	✓	✓
Mixed	Homozygous Reference (Derived)	✓	✓
Pug	Homozygous Reference (Derived)	✓	✓
Pug	Homozygous Reference (Derived)	✓	✓
Shih Tzu	Homozygous Reference (Derived)	✓	✓
Airedale Terrier	Homozygous Variant (Ancestral)	✓	✓
Italian Greyhound	Homozygous Variant (Ancestral)	✓	✓
Saluki	Homozygous Variant (Ancestral)	✓	✓
Whippet	Homozygous Variant (Ancestral)	✓	✓
Wolf	Homozygous Variant (Ancestral)	✓	X
Wolf	Homozygous Variant (Ancestral)	✓	X
Wolf	Homozygous Variant (Ancestral)	✓	X
Wolf	Homozygous Variant (Ancestral)	✓	X

Table S4. **Filtering Criteria.** Related to Table 2. Criteria for hard filtering variants within the critical interval (CFA1:55,850,299 – 56,037,676) were determined by the presence or absence of the derived twelve-SNP haplotype. A variant must satisfy each criteria in order to remain as a candidate for SnpSift or Pindel filtering.

Transcript	Silent Variant (55,939,143)	Splice Donor			Splice Acceptor			RNAseq Transcript Count								
		Position	Feature	Sequence	Sequence	Feature	Position	BULD	FBUL	PUG-	CKCS	CKCS	PAPI	ITGY	WHIP	YORK
Canonical (Ancestral)	AAT GA**C/T** AAC	55,939,268	Exon 8	TCC ACC AG **gt**aagg	tcgt**ag** G TAC GAA	Exon 9	55,947,842	224	84	116	258	1012	361	493	644	911
Splice Variant 1	AAT GA**T** AAC	55,939,268	Exon 8	TCC ACC AG **gt**aagg	gctc**ag** C CCT CAG	Intron 8	55,939,358	5 (2.23)	0 (0)	0 (0)	0 (0)	0 (0)	0 (0)	0 (0)	0 (0)	0 (0)
Splice Variant 2	AAT GA**T** AAC	55,939,268	Exon 8	TCC ACC AG **gt**aagg	cctc**ag** A CCT CAC	Intron 8	55,939,365	0 (0)	0 (0)	0 (0)	0 (0)	0 (0)	2 (0.55)	0 (0)	0 (0)	0 (0)
Splice Variant 3	AAT GA**T** AAC	55,939,268	Exon 8	TCC ACC AG **gt**aagg	ggac**ag** T GCT GGC	Intron 8	55,939,387	0 (0)	0 (0)	0 (0)	0 (0)	2 (0.19)	0 (0)	0 (0)	0 (0)	0 (0)
Splice Variant 4	AAT GA**T** AAC	55,939,268	Exon 8	TCC ACC AG **gt**aagg	ctgc**ag** G TTC TGG	Intron 8	55,939,436	87 (38.83)	17 (20.23)	43 (37.06)	9 (3.48)	40 (3.95)	13 (3.60)	0 (0)	0 (0)	1 (0.10)
Splice Variant 5	AAT GA**T** AAC	55,939,268	Exon 8	TCC ACC AG **gt**aagg	atac**ag** G CTG GCA	Intron 8	55,939,566	2 (0.89)	1 (1.19)	4 (3.44)	1 (0.38)	3 (0.29)	0 (0)	2 (0.40)	0 (0)	0 (0)
Splice Variant 6	AAT GA**T** AAC	55,939,268	Exon 8	TCC ACC AG **gt**aagg	tttc**ag** C AAA GGG	Intron 8	55,939,658	20 (8.92)	7 (8.33)	11 (9.48)	1 (0.38)	5 (0.49)	0 (0)	0 (0)	0 (0)	0 (0)

Table S5. Splice Site Sequences for *SMOC2* Isoforms. Related to Figure 4. Upper-case letters indicate sequence included in mRNA. Lower-case letters indicate intron sequence that is not included in mature mRNA transcripts. Bold letters are the consensus splice donor (gt) and splice acceptor (ag) sites. Three dogs for each *SMOC2* LINE-1 genotype status were used for RNAseq analysis – homozygous derived (Bulldog, French Bulldog, Pug) heterozygous (Cavalier King Charles Spaniel x2, Papillion) and homozygous ancestral (Italian Greyhound, Whippet, Yorkshire Terrier). The percentage of splice variant read count of the canonical transcript count for each dog are given in brackets.

Individual	Breed	*SMOC2* Genotype	Silent Variant (55,939,143)	
			T-allele	C-allele
1	BULD	DD	100%	
2	FBUL	DD	100%	
3	PUG-	DD	100%	
4	PAPI	AD	100%	
5	CKCS	AD	24%	76%
6	CKCS	AD	25%	75%
7	ITGY	AA	100%	
8	WHIP	AA	100%	
9	YORK	AA	54%	46%

Table S6. Allele-specific Expression of *SMOC2*. Related to Figure 4. RNAseq data was generated for nine dogs for the homozygous derived (D/D), heterozygous (AD) and homozygous ancestral (AA) genotypes of *SMOC2*. The sequencing of the C/T silent mutation in exon 8 is given as a percentage of the total read counts for the 55,939,143 base position. The LINE-1 element is found on a T-allele which suggests the decreased abundance of transcripts from the LINE-1 carrying haplotype.

Supplemental References

S1. Hayward, J. J., Castelhano, M. G., Oliveira, K. C., Corey, E., Balkman, C., Baxter, T. L., Casal, M. L., Sharon A Center, Fang, M., Garrison, S. J., et al. (2016). Complex disease and phenotype mapping in the domestic dog. Nat Commun *7*, 10460.

S2. Boyko, A. R., Quignon, P., Li, L., Schoenebeck, J. J., Degenhardt, J. D., Lohmueller, K. E., Zhao, K., Brisbin, A., Parker, H. G., vonHoldt, B. M., et al. (2010). A simple genetic architecture underlies morphological variation in dogs. PLoS Biol *8*, e1000451.

S3. Rimbault, M., Beale, H. C., Schoenebeck, J. J., Hoopes, B. C., Allen, J. J., Kilroy-Glynn, P., Wayne, R. K., Sutter, N. B., and Ostrander, E. A. (2013). Derived variants at six genes explain nearly half of size reduction in dog breeds. Genome Res *23*, 1985–1995.

S4. Parker, H. G., vonHoldt, B. M., Quignon, P., Margulies, E. H., Shao, S., Mosher, D. S., Spady, T. C., Elkahloun, A., Cargill, M., Jones, P. G., et al. (2009). An expressed fgf4 retrogene is associated with breed-defining chondrodysplasia in domestic dogs. Science *325*, 995–998.
